# Obg-Like ATPase 1 Enhances Chemoresistance of Breast Cancer *via* Activation of TGF-β/Smad Axis Cascades

**DOI:** 10.3389/fphar.2020.00666

**Published:** 2020-05-27

**Authors:** Jianzhou Liu, Xiaoyu Miao, Bowen Xiao, Jing Huang, Xufeng Tao, Jiong Zhang, Hua Zhao, Yue Pan, Hongwei Wang, Ge Gao, Gary Guishan Xiao

**Affiliations:** ^1^State Key Laboratory of Fine Chemicals, Department of Pharmaceutical Sciences, School of Chemical Engineering, Dalian University of Technology, Dalian, China; ^2^School of Bioengineering, Dalian University of Technology, Dalian, China; ^3^Cardiothoracic Surgery, Changsha Central Hospital Affiliated to Nanhua University, Changsha, China; ^4^Beijing Key Lab of Plant Resource Research and Development, Beijing Technology and Business University, Beijing, China; ^5^Department of Dermatology, Peking Union Medical College Hospital, Beijing, China; ^6^Department of Laboratory Medicine, The Third Xiangya Hospital, Central South University, Changsha, China; ^7^Functional Genomics and Proteomics Laboratory, Osteoporosis Research Center, Creighton University Medical Center, Omaha, NE, United States

**Keywords:** Obg-like ATPase 1, breast cancer, chemosensitivity, γ-tubulin, multidrug resistance, paclitaxel, 5-FU

## Abstract

Understanding the molecular mechanism of drug resistance helps to identify an effective target for breast cancer therapy. In this study we investigated the regulatory role of Obg-like ATPase 1 which is involved in multiple uses of drug resistance against breast cancer. Paclitaxel resistant cell line (MCF-7-PTR) was developed by a continuous increasing paclitaxel concentration. MTT assay was used to validate either acquired resistant or OLA1 modified cell lines. qRT-PCR, western blotting, apoptosis, and cell cycle assays were executed to evaluate gene and protein expression in cell lines. A series of *in vitro* assays was performed in the cells with RNAi-mediated knockdown to expound the regulatory function of OLA1 in breast cancer. We demonstrated that OLA1 was highly correlated with either acquired or intrinsic resistance of breast cancer. Further study showed that escalated expression of OLA1 promoted the EMT process in tumor cells through TGF-β/Smad signaling cascades, resulting in the enhanced expression of anti-apoptosis-related proteins (cleaved caspase3, Bax, Bcl-2) and the strengthening depolymerization of microtubules in tumor cells. Our findings revealed that OLA1 enhanced the anti-apoptotic ability and elucidated a regulatory role of OLA1 in promoting chemotherapy resistance of breast cancer. Chemo-sensitivity of the disease can be thus enhanced significantly by knocked down OLA1, which led to the inactivation of the TGF-β/Smad signaling cascades, polymerized microtubules, and promoted cell apoptosis. Our data suggest that OLA1 may be developed as a potential target to improve chemotherapy of patients with breast cancer.

## Introduction

Breast cancer is one of the leading fatal cancers among women worldwide. According to statistics, there were an estimated 2.1 million breast cancer patients globally in 2018, and one out of every four female cancer patients is a breast cancer patient (Bray et al., 2018). Although breast cancer is a relatively easily diagnosed cancer in most countries, this does not prevent it from being a most lethal disease. The resistance of tumor cells to chemotherapy is still a critical issue breast cancer therapy.

Epithelial-to-mesenchymal transition (EMT), which is associated with the acquisition of stem cell properties, metastasis, and resistance to therapy (Mani et al., 2008; Pastushenko et al., 2018), is a process in which the characteristics of epithelial cells change to the mesenchymal phenotype. Reasonably, breast cancer that remains after chemotherapy shows mesenchymal phenotypes and tumor initiation characteristics (Creighton et al., 2009). In fact, the evidence that EMT promotes tumor metastasis is insufficient, but EMT can indeed promote tumor chemoresistance in various cancers (Fischer et al., 2015; Zheng et al., 2015; Diepenbruck and Christofori, 2016; Ye et al., 2017). Emerging evidence has proven that EMT makes tumor cells more chemoresistance when cells are transfected with specific hallmark genes of EMT, including Wnt and the transforming growth factor-β (TGF-β) (Cufi et al., 2010; Li et al., 2013; Mallini et al., 2014). Actually, the TGF-β pathway has a crucial role in EMT induction in a variety of tissue types (Xu et al., 2009; Lamouille et al., 2014). Adding TGF-β to culture mediums of epithelial cells is a convenient method to induce EMT (Xu et al., 2009). Exposure of tumor cells to TGF-β and TNF-α induces the EMT process and generates cells with a cancer stem cell-like phenotype, which is shown by the increased self-renewal capacity resulting in greatly improved tumorigenicity, and enhanced resistance to oxaliplatin, etoposide, and paclitaxel (Asiedu et al., 2011).TGF-β signaling possesses both Smad and non-Smad pathways, and crosstalk with numerous signal transduction pathways to advance EMT processes at multiple levels, including PI3K-AKT-mTOR, Wnt, Notch, and ERK, p38, and JUN N-terminal kinase (JNK) MAPK pathways (Xu et al., 2009; Lamouille et al., 2014). A study showed that breast cancer MDA-231 cells treated with cisplatin increased TGF-β mRNA expression. When TGF-β neutralizing antibodies were used to block the activity of TGF-β in tumor cells, breast cancer cells resumed sensitivity to the drug (Ohmori et al., 1998). Moreover, MCF-7/tamoxifen-resistant cells experienced the EMT process driven by an intensive endogenous TGF-β/Smad signaling pathway. Ectopic supplements of TGF-β promoted a mesenchymal transition of MCF-7 cells showing a resistant phenotype (Shi et al., 2013).

Paclitaxel (PTX) is a microtubule-stabilizing agent which is approved by the Food and Drug Administration(FDA) for the therapy of ovarian, breast (Gampenrieder et al., 2013; Zardavas and Piccart, 2015), and lung cancer, as well as leukaemias and lymphomas (Wertz et al., 2011). Paclitaxel in general induces mitotic arrest to mediate arrested cell death. Microtubules play pivotal roles in basic cellular processes and are targets used for anti-tubulin chemotherapeutics (Wertz et al., 2011). Microtubules are composed of tubulin monomers joined together by non-covalent bonds. There are two subunits of tubulin: α-tubulin and β-tubulin. Either assembly or disassembly of tubulin is highly relied on in cellular GTP and GDP (Muller-Reichert et al., 1998; Wang and Nogales, 2005; Alushin et al., 2014). γ-tubulin, not a component of microtubules, is involved in the assembly of microtubules (Oegema et al., 1999). It is important in the nucleation and polar orientation of microtubules (Joshi et al., 1992). It is mainly found in centrosomes and spindle poles because they are the most abundant microtubule nucleation areas (Wolf and Joshi, 1996).

Obg-like ATPase 1(OLA1) is a p-loop GTPase belonging to the TRAFAC (translation factor related) class, the Obg family and the YchF subgroup. The main functions of TRAFAC GTPase include: translation factors and ribosomal connexin, signal transduction, intracellular transport, and stress response proteins (Leipe et al., 2002; Verstraeten et al., 2011). OLA1 is highly conserved from bacteria to humans, and unlike other Obg family members, exercises both GTPase and ATPase activities (Koller-Eichhorn et al., 2007; Gradia et al., 2009). OLA1 was a DNA damage related and cell growth regulated gene, and decreased cellular sensitivity to doxorubicin in colon cancer cell (Sun et al., 2010). Currently, literature reports that OLA1 was involved in EMT transformation in different tumor cells (Zhang et al., 2009b; Bai et al., 2016). EMT is not a prerequisite for metastasis but contributes to chemoresistance (Fischer et al., 2015). However, whether OLA1 also mediates the EMT process in drug-resistant cells is not known yet. Thus far, there is no report showing whether OLA1 is associated with breast cancer drug resistance. Recent studies showed that OLA1 can also interact with γ-tubulin and form a complex with breast cancer 1 (BRCA1) and BRCA1-associated RING domain protein (BARD1), leading to the recruitment of receptors for activated C kinase 1 (RACK1) to regulate the centrosome (Matsuzawa et al., 2014; Yoshino et al., 2018; Yoshino et al., 2019). We hypothesize that OLA1 may regulate paclitaxel resistance of breast cancer by interfering tubulin expression. In this study, we show that OLA1 is positively correlated with the development of drug resistance by inducing the EMT process through activation of TGF-β/Smad signaling pathway in breast cancer. Our results indicate OLA1 can be developed as a novel valuable target for an improvement of breast cancer chemotherapy.

## Materials and Methods

### Materials

PTX (#S1150) and Fluorouracil (5-FU, #S1209) were purchased from Selleck Chemicals LLC (Shanghai, China). Antibodies against OLA1 (also as GTPBP9, #PA5-31227) and Snail (#PA5-11923) were purchased from Invitrogen (San Diego, CA, USA). Antibodies against GAPDH (#5174), Slug (#9585P), Vimentin(#3932S), Smad3 (#9523P), p-Smad3 (#9520P), Smad4 (#9515P), Cleaved caspase3 (#9661), Bax (#2772), and Bcl2 (#2876S) were purchased from Cell Signaling Technology (Danvers, MA, USA). Antibodies against gamma tubulin (#ab11316), Zeb1 (#ab180905) and E-cadherin (#ab76055) were purchased from Abcam (Cambridge, MA, USA). GAPDH was purchased from ProteinTech Inc (Wuhan, China). The second antibodies were purchased from Abbkine Inc (Wuhan, China). SuperEnhanced chemiluminescence (ECL) detection reagents and RIPA lysis buffer were purchased from Applygen Technologies Inc. (Beijing, China). Apoptosis Kit was purchased from KeyGEN BioTECH Inc (Nanjing, China). (4,5-dimethylthiazol-2-yl)-2,5-diphenyltetrazolium bromide (MTT) were obtained from Solarbio Inc (Beijing, China). All other reagents were from Beyotime (Haimen, China) and Sangon Biotech (Shanghai, China).

### Cell Culture

Human MCF-7 breast carcinoma cells were kindly provided by Stem Cell Bank, Chinese Academy of Sciences(Shanghai, China), and routinely grown in Dulbecco’s modified Eagle’s medium (DMEM; Gibco, Cat#12430054) containing 10% fetal bovine serum (PAN, Germany) and a mixture of 100 IU/ml penicillin and 100 μg/ml streptomycin (Solarbio, Beijing, China) in an incubator at 37 °C, 5% CO_2_. The paclitaxel-resistant sublines (MCF-7-PTR) were derived from MCF-7 by continuous exposure to PTX. MCF-7-PTR cells were cultured continuously in a medium containing 10% FBS supplemented with 100 nM PTX. MDA-MB-231 were kindly provided by Dr. Zhengzheng Shi, and the culture conditions were similar with MCF-7 as described above.

### Cell Viability Assay

MTT assay was used to determine the viability of the treated cells. Digest, collect, and count cells and 6000 cells were seeded onto 96-well plates and incubated overnight at 37°C. Paclitaxel at different concentrations (0, 1.56, 3.13, 6.25, 12.50, 25.0, 50.0, and 100.0 μM) were added to each well with different incubation times. Thereafter, 20 μL MTT solution (5 mg/mL) was added to each individual well. After incubating for 4 h at 37°C, the media was aspirated and 150 μL DMSO was added to dissolve the formazan crystals. The absorbance was measured at 490nm by the microplate reader. Six replicate wells were included in each analysis and at least three independent experiments were conducted. The cell inhibition rate and IC50 were calculated respectively by SPSS. The same method was used for the measurement of 5-Fu.

### Cell Proliferation

Cell proliferation ability was assessed using an MTT assay. The silence group was transfected with either siOLA1 or silence negative control (siNC) for 12h before use. Cells were seeded at a density of 2 × 10^3^ cells/well in 96-well plates. In the experimental group transfected with interfering RNA, the culture medium containing the transfection reagent and the interfering RNA was replaced once on the fourth day of the experiment. Cells, on day 1, 2, 3, 4, 5, 6, and 7 were incubated with 20 μL MTT solution at 37°C for 4 h. With the incubation medium removed, 150 μL DMSO was added to each well. The absorbance was measured at 490 nm.

### Construction of Paclitaxel Resistant Cell Lines

Low-concentration induction method was used to construct drug-resistant cell lines. Briefly, the IC50 of paclitaxel on MCF-7 was detected, and the appropriate initial concentration (100 nM) was selected and added into the culture flask for incubation. After 24 h, the medicated medium was aspirated, washed with PBS, and added to the normal medium until the cells were over 80% and passaged. The medium was incubated n with equal concentration of the drug depending on cell growth, or a four-fold dose of the drug was added. This process was cycled back and forth until the resistance met the experimental needs. The establishment cycle of acquired drug-resistant cells was about six months.

### Small Interfering RNA Transfections

Small interfering RNA (siRNA) for OLA1 (Cat#EHU113781) and siRNA Universal Negative Control #1 (Cat#SIC001) were purchased from Sigma-Aldrich. Cells seeded in a 6-well plate were transiently transfected with 100 pM siRNA with Lipo2000 Transfection Reagent (Thermo Scientific) according to manufacturer’s instructions.

### Establishment of the Stable OLA1 Knockdown MDA-MB-231 Cell Lines.

Small hairpin RNA (shRNA) lentiviral used for stable silencing of OLA1 (shOLA1) and the control non-targeting plasmid (shNC) were purchased from GenePharma (Nanjing, China) by inserting the following short-hairpin sequences into the pGLV3/H1/GFP/Puro vector:

5′-CCGGGAGGAAATGATTGGGCCCATTCTCGAGAATGGGCCCAAT CATTTCCTCTTTTTTG-3′ for sh-OLA1 and 5′-CCGGCAACAAGATGAAGAG CACCAACTCGAGTTGGTGCTCTTCATCTTGTTGTTTTTG-3′ for Small hairpin control (shNC). shRNA transfections and protocol were followed the recommendations by GenePharma (China). The shNC and shOLA1 vectors were transfected into MDA-MB-231 cells. The knockdown efficiency of the target gene was verified by qRT-PCR and western blot analysis.

### qRT-PCR Analysis

Total RNA was extracted from the cells using TRIzol (Cat#15596026, Invitrogen, CA, USA), and the concentration and quality were determined by a microplate reader (DU730, Beckman, CA, USA). The nucleotides were reverse-transcribed into cDNA according to the instructions of the PrimeScript™ RT Reagent Kit (Cat#RR037A, Takara, Japan). After amplification and dilution, the assay was performed on the *LightCycler480 II* (Roche, USA). The gene primer was as follows: GAPDH (glyceraldehyde 3-phosphate dehydrogenase): Forward primer: 5-CATGAGAAGTATGACAACAGCCT Reverse primer: 5-AGTCCTTCCACGATACCAAAGT; OLA1: Forward primer: 5-TGGACAAGTATGACCCAGGT Reverse primer: 5-GCTGCAAACCCAGCCTTAATG. The other primer sequences are provided in the supplemental material (**Supplementary Table 1**).

### Western Blotting Analysis

Protein was extracted from the cells using RIPA buffer, added with PMSF to avoid degrading, and stored at -80°C. The BCA protein concentration detection kit was used for quantification, and the loading buffer was added in proportion to boil at 95 °C and stored in a refrigerator at -20 °C. SDS-PAGE gel was prepared and 30 μg of protein sample was added to each lane. The target protein band was cut and transferred to the PVDF membrane, and the milk was blocked for 2 h. The membrane was washed three times with TBST (10 min/time), added with a primary antibody at 4°C overnight, then washed three times with TBST (10 min/time), and the secondary antibody was incubated for 2 h. After TBST washing, the membrane was incubated with ECL high-sensitivity developer and then developed in ChemiDoc Imaging Systems (BIO-RAD, USA).

### Apoptosis Analysis

Cells (2 × 10^5^) were seeded onto 6-well plates for each group overnight then treated with paclitaxel (20 μM) for the indicated time. After incubation, the medium was collected, and the cells were digested with trypsin without EDTA and incorporated into the previously collected medium, where total cells were collected by centrifugation. Following the steps of Annexin V-FITC/PI double staining kit, staining reagents were added twice in turn, incubated at room temperature for 10 min in the dark, and then apoptosis analysis was performed by flow cytometry.

### Cell Cycle Analysis

Cells were stained with propidium iodide (PI) using the cell cycle kit (#KGA511, KeyGEN BioTECH, Nanjing, China) according to the provided protocol. Briefly, cells were harvested, washed in ice-cold phosphate-buffered saline (PBS), and fixed in 70% cold ethanol for 2h at 4°C. After two PBS washes, cells were treated with RNase A/PI staining buffer and assayed with an FACS Calibur (BD Biosciences, San Jose, CA, USA) flow cytometer using Cell Quest software. The cell cycle distribution was analyzed using BD CellQuest™ Pro Analysis software (BD Biosciences, San Jose, CA, USA).

### Statistical Analysis

Data were presented as mean ± standard deviation (SD). IC50 (mean ± 95% confidence interval) of chemotherapeutics in breast cancer was calculated by SPSS23.0, and other statistic results were carried by GraphPad Prism 8. A two-sided tail non-paired Student’s t test was used to compare the differences of two groups. Kaplan-Meier analysis and logrank test was used to assess statistical significance of survival rate. *P <*0.05 was considered statistically significant.

## Results

### OLA1 Was Upregulated in Breast Cancer

**Figure 1:**
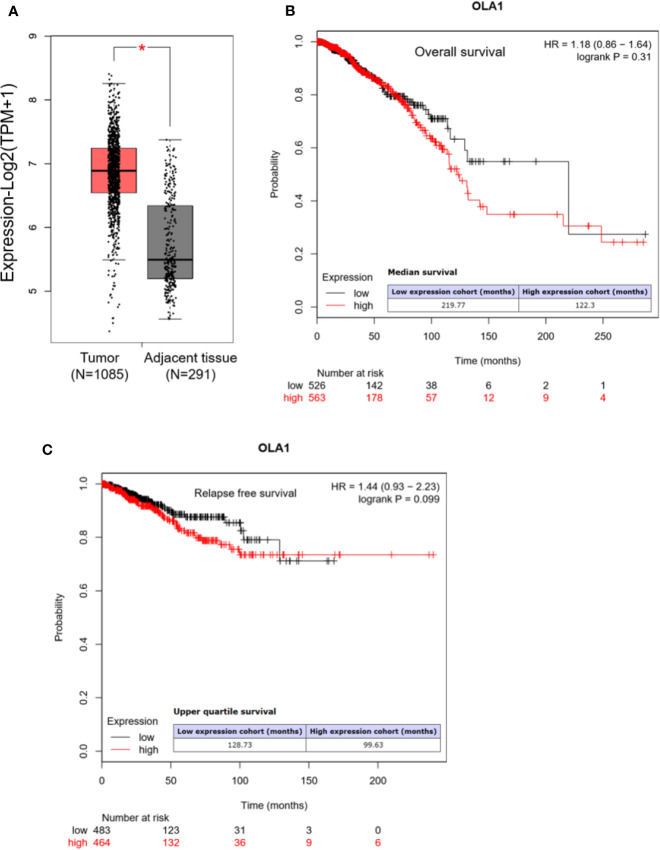
OLA1 is upregulated in breast cancer. **(A)** The average expression level of OLA1 in patient’s breast cancer tissue was higher than adjacent tissues in TCGA and GTEx breast cancer dataset. **(B, C)** Kaplan-Meier overall survival and relapse free survival curves for patients with breast cancer stratified by high and low expression of OLA1.

We first analyzed OLA1 expression profile across all tumor samples and paired normal tissues in the RNA sequencing data from Gene Expression Profiling Interactive Analysis (GEPIA) (Tang et al., 2017). We found that OLA1 has a ubiquitous expression in the brain, thyroid, and 25 other tissues from the body map (**Supplementary Figure 1A**), and is highly expressed in breast cancer, pancreatic cancer, colorectal cancer, and other cancer tissues (**Supplementary Figure 1B**). Through matching with The Cancer Genome Atlas (TCGA) normal and Genotype-Tissue Expression (GTEx) data, we found that OLA1 is significantly upregulated in breast cancer (N=1085) compared to their paired normal tissue (N=291) (**Figure 1A**). Notably, the Kaplan-Meier (KM) Plotter analysis (Nagy et al., 2018) showed that OLA1 expression may negatively correlate with the overall survival and relapse free survival of breast invasive carcinoma (BRCA) patients (**Figures 1B, C**). Although the *P*-value is more than 0.05, the survival time with OLA1 highly expressed cohort is much less than the lower, suggesting OLA1 may play a regulatory role in drug resistance.

### Upregulated OLA1 Was Also Observed in Acquired Drug-Resistant Cell Line MCF-7-PTR

**Figure 2:**
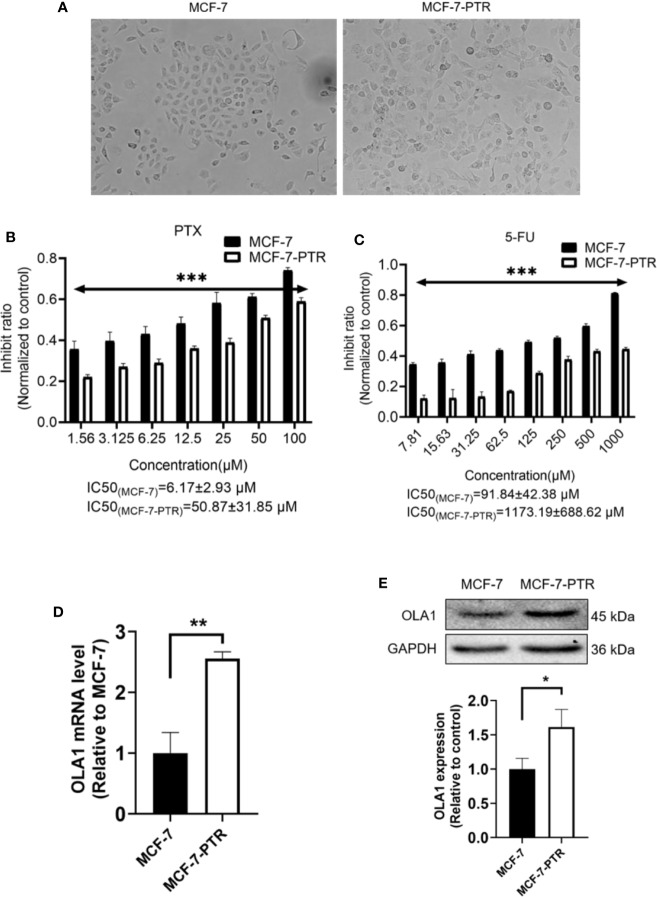
Upregulated of OLA1 in acquired drug-resistant cell line MCF-7-PTR. **(A)** Morphology of paclitaxel-induced MCF-7-PTR cells and the parent MCF-7 cells (100X). **(B)** Drug resistance assay for enhanced expression of OLA1 promotes MCF-7-PTR cell resistance to PTX. **(C)** Drug resistance assay for enhanced expression of OLA1 promotes MCF-7-PTR cell resistance to 5-Fu. MCF-7 cells and MCF-7-PTR cells were analyzed for the presence of OLA1 by RT-PCR **(D)**, Western blotting **(E)**. The relative fold-change was compared with MCF-7 cells (*P < 0.05, **P <0.01, ***P < 0.001, Student’s t-test).

To understand the regulatory role of OLA1 in breast cancer resistance to PTX, MCF-7 resistant cell line to PTX was developed (named as MCF-7-PTR) through continuous induction of MCF-7 with PTX at a low concentration (100 nM). To understand whether multiple drug resistance of MCF-7-PTR was also conferred, the resistance of MCF-7-PTR to 5-Fu was also examined and showed that multiple drug resistance of breast cancer cells were formed, as shown in **Figure 2**. MTT assays showed that the IC50 of MCF-7-PTR to paclitaxel was 50.87 ± 31.85 μM compared to the parental cells MCF-7 (IC50 6.17 ± 2.93 μM), and the drug resistance index was 8.24 (**Figure 2B**). The IC50 of MCF-7-PTR to 5-Fu was 1173.19 ± 688.62 μM as compared to the parental cells MCF-7 (i.e. 91.84 ± 42.38 μM), and the drug resistance index was 12.77 (**Figure 2C**). During development of the drug antagonistic to breast cancer, cell morphology was changed significantly, observed to be more dispersed and irregular in resistant cells than that of the parental cells (**Figure 2A**). To find out whether endogenous OLA1 is related to the development of drug resistance in breast cancer, the endogenic level of OLA1 in both mRNA (**Figure 2D**) and protein levels (**Figure 2E**) in MCF-7-PTR was analyzed, and showed that both endogenous levels of mRNA and protein of OLA1 were indeed significantly higher than that of the parental cells (*P< 0.05, **P < 0.01), indicating that OLA1 plays a regulatory role in the development of tumor drug resistance.

### Knockdown of OLA1 Enhanced Chemo-Sensitivity of the Acquired Drug Resistance of Breast Cancer

**Figure 3:**
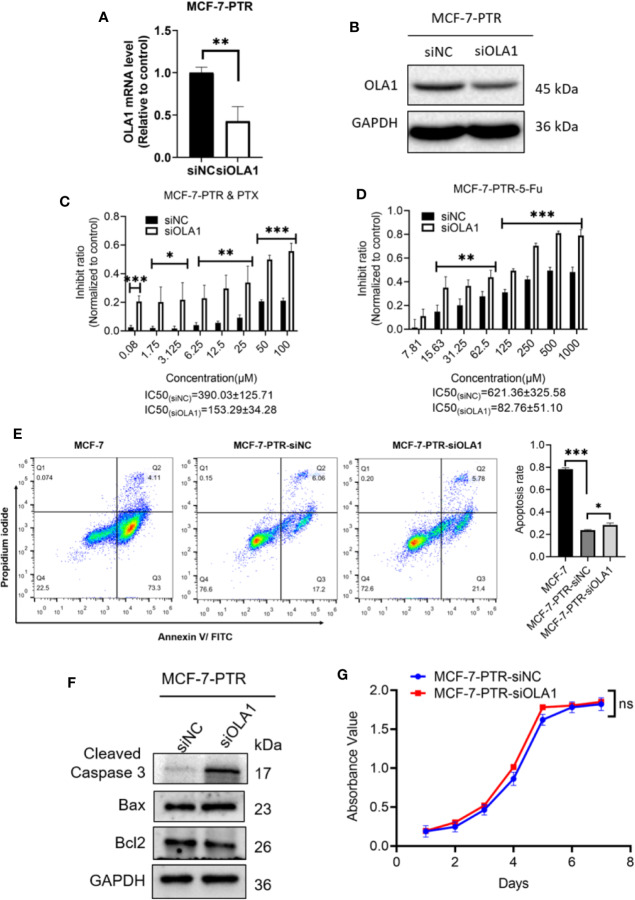
Knockdown of OLA1 suppress the chemoresistance and enhanced chemo-sensitivity of acquired drug resistance cell in breast cancer. **(A, B)** were mRNA and protein levels of OLA1 with or without siOLA1 in MCF-7-PTR. **(C, D)** Depletion of OLA1 enhances the PTX or 5-Fu sensitivity of MCF-7-PTR cells by specific siRNA respectively. (*P < 0.05, **P < 0.01, ***P < 0.001, Unpaired Student’s t-test.) **(E)** Annexin V-FITC and PI staining of the indicated cells treated with PTX (20 μM) for 24 h (apoptosis rate is the sum of early and late apoptosis rates). (*P< 0.05, ***P < 0.001, Unpaired Student’s t-test.) **(F)** Western blotting analysis of cleaved caspase3, Bax, and Bcl2 in the indicated cells; GAPDH was used as a loading control. **(G)** Cell proliferation curve was drawn in the indicated cells. ns, no significance.

To further determine the regulatory role of OLA1 in drug resistance, small interfering RNA of OLA1 was successfully used to knockdown the endogenic level of OLA1 in MCF-7-PTR, as shown in both mRNA and protein levels (**Figures 3A, B**). Acquired resistant cell MCF-7-PTR regained its sensitivity to paclitaxel after knocking down of the endogenous OLA1 (*P < 0.05, **P < 0.01, ***P < 0.001) (**Figures 3C, D**), as shown in the flow cytometry analysis of MCF-7-PTR (**Figure 3E**). To further confirm apoptosis induced by siRNA-OLA1 treatment, the expression level of Bcl-2 and the proapoptotic protein Bax and apoptosis terminal factor Caspase-3 were examined and showed that Bcl-2 was significantly decreased while Bax and Caspase-3 were increased remarkably (**Figure 3F**). Intriguingly, regardless of whether OLA1 was silenced or not, no significant effect on cell proliferation of MCF-7-PTR was observed (**Figure 3G**).

### Knockdown of OLA1 Enhanced Chemo-Sensitivity of Intrinsic Drug Resistance of Breast Cancer

**Figure 4:**
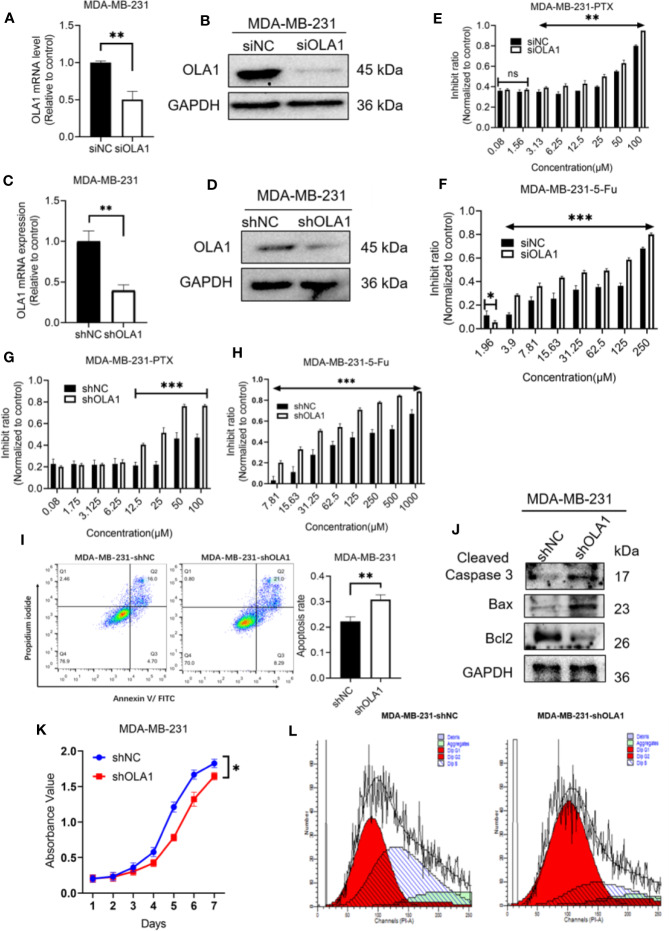
Knockdown of OLA1 suppress chemoresistance and enhanced chemo-sensitivity of endogenous drug resistance cell in breast cancer. **(A, B)** were mRNA and protein level of OLA1 with or without siOLA1 in MDA-MB-231. **(C, D)** were mRNA and protein level of OLA1 with or without shOLA1 in MDA-MB-231. **(E, F)** Depletion of OLA1 enhances the PTX or 5-Fu sensitivity of MDA-MB-231 cells by specific siRNA respectively. **(G, H)** Knockdown of OLA1 enhances the PTX or 5-Fu sensitivity of MDA-MB-231 cells by short hairpin RNA. **(I)** Annexin V-FITC and PI staining of the indicated cells treated with PTX (20 μM) for 24 h. **(J)** Western blotting analysis of cleaved caspase3, Bax and Bcl2 in the indicated cells; GAPDH was used as a loading control. **(K)** Cell proliferation curve was drawn in indicated cells. **(L)** The cell-cycle distribution was assessed following transduction by flow cytometry. Data represent the mean ± SD from three independent experiments.(*P < 0.05 **P < 0.01, ***P < 0.001, Student’s t-test).

To understand whether endogenous OLA1 is also associated with intrinsic drug resistance of breast cancer cells, a triple-negative breast cancer cell line MDA-MB-231 was utilized. The endogenic level of OLA1 in MDA-MB-231 cells was significantly higher than MCF-7 (**Supplementary Figure 1C**), while it can be remarkably suppressed if it was silenced (**Figures 4A, B**). To understand whether knocked down OLA1 in MDA-MB-231 can enhance the chemosensitivity of the triple negative breast cancer, Knocked down of OLA1 stably in MDA-MB-231 was performed (**Figures 4A, B**), and it was found that the expression of OLA1 was meaningfully decreased in the transfected group compared with the control group (**P < 0.01) (**Figures 4C, D**). The sensitivity to paclitaxel of MDA-MB-231 cells in the knockdown OLA1 group was not changed at low doses of the drug, but significantly enhanced at high doses of the drug (**Figures 4E–H**). The enhanced sensitivity to paclitaxel of MDA-MB-231 was further confirmed by analysis of flow cytometry when OLA1 was knocked down as shown in **Figure 4I**. Decreased level of Bcl-2 and the increased levels of Bax and Caspase-3 were also observed (**Figure 4J**), suggesting that knock down of OLA1 also promoted the sensitivity of breast cancer cells with intrinsic resistance to chemotherapy. This notion was further confirmed by retarded cell proliferation (**Figure 4K**), which was supported by an arrested activity in the cell cycle indicated by a blockage at the G1/S phase (**Figure 4L**) and the reduced CCND1 (**Figure 6D**). However, there was no change observed in the expression of CCND1 in MCF-7-PTR (**Figure 6C**), consistent with the cell growth, suggesting that there may be other interactive factors involved in OLA1 signaling cascade.

### Downregulation of OLA1 Inhibited EMT Progression in Drug-Resistant Breast Cancer Cell Lines

**Figure 5:**
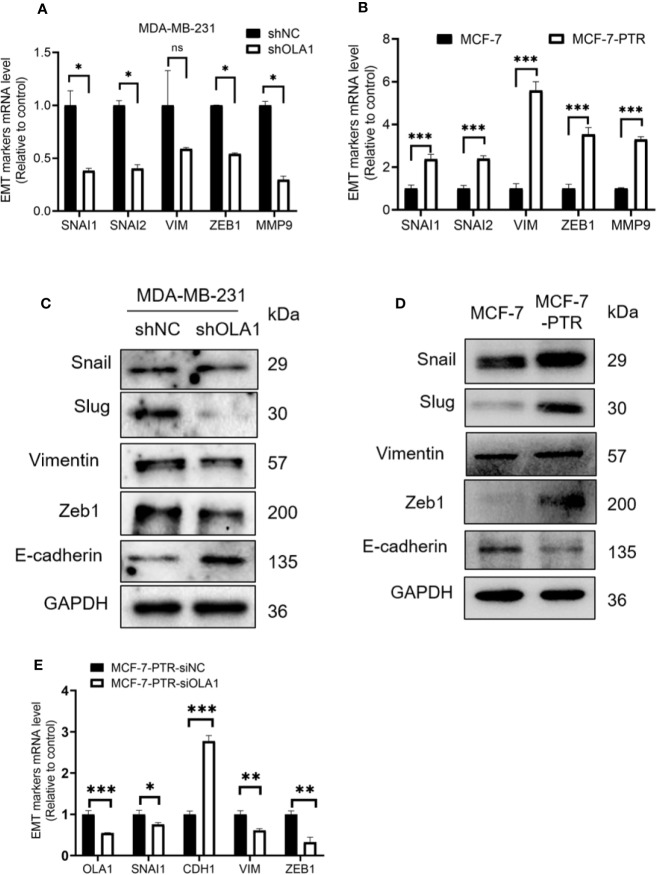
Down regulated OLA1 inhibits EMT progress in drug-resistant cell lines. **(A)** The effect of OLA1 knockdown on expression of Snail, Slug, Vimentin, Zeb1, and MMP9 were evaluated by qRT-PCR. **(B)** mRNA levels of Snail, Slug, Vimentin, Zeb1, and MMP9 in MCF-7-PTR were calculated comparing with MCF-7. **(C)** The effect of OLA1-KD on E-cadherin, Vimentin, and Snail 1, Slug, and Zeb1 protein levels were confirmed by western blotting. **(D)** Protein levels of E-cadherin, Vimentin, and Snail 1, Slug, and Zeb1 in MCF-7-PTR were calculated comparing with MCF-7. **(E)** mRNA levels of OLA1, Snail, E-cadherin, Vimentin, and Zeb1 in MCF-7-PTR-siOLA1 were calculated comparing with siNC. (*P < 0.05, **P < 0.01, ***P < 0.001, Student’s t-test). ns, no significance.

EMT is a biological process that allows polarized epithelial cells to undergo a variety of biochemical changes, making them exhibit a mesenchymal cell phenotype, including increased migratory capacity, invasiveness, and enhanced anti-apoptotic capabilities. To understand the molecular mechanism underlying OLA1-mediated drug resistance to breast cancer cells, biomarkers in the EMT process were analyzed. The results showed that knocked down OLA1 in the MDA-MB-231-shOLA1 decreased the expression of Snail, MMP9, Vimentin, Slug, zeb-1 (*P* < 0.05) (**Figures 5A, C**), and incremental E-cadherin (**Figure 5C**). We also found that the expression of Snail, MMP9, Vimentin, Slug, and Zeb-1 was significantly increased in MCF-7-PTR cells (*P* < 0.05) (**Figures 5B, D**) and decreased E-cadherin, suggesting that OLA1 may regulate EMT process positively. To understand the effects of chemoresistance of MCF-7 cells with the effects of the extinction of OLA1 in MDA-MB-231 cells, SNAI1, VIM, ZEB1, and CDH1 in MCF-7 PTR cells transfected with siOLA1 were detected (**Figure 5E**). The results showed that knocked down OLA1 in the MCF-7-PTR decreased the EMT process.

### OLA1 Induced EMT Phenotype *via* TGF-β/Smad Pathway in Breast Cancer

**Figure 6:**
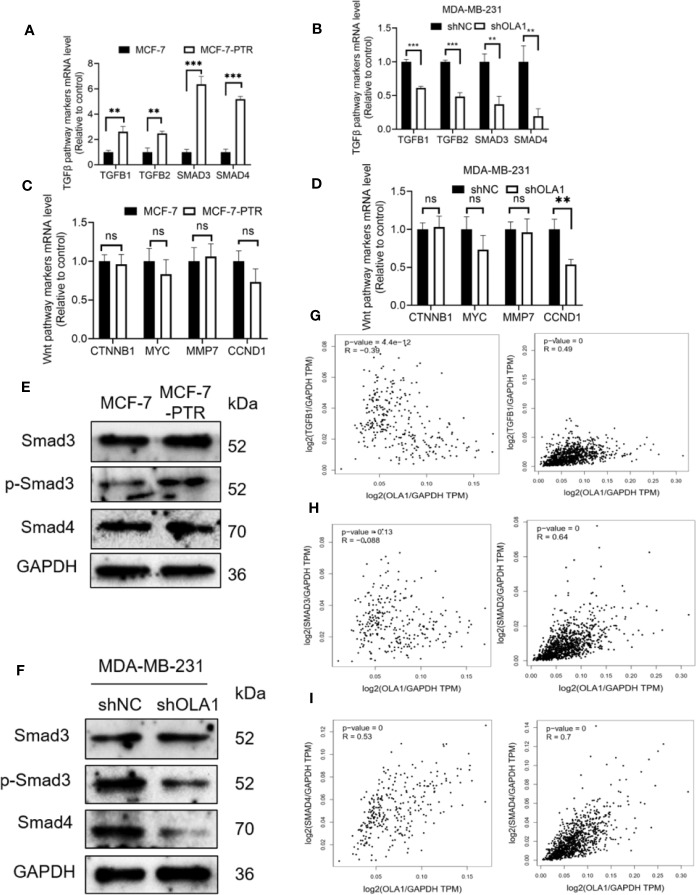
OLA1 induces EMT phenotype by TGFβ/Smad pathway in breast cancer. TGFβ/Smad signaling marker mRNA level was confirmed in MCF-7-PTR **(A)**, and MDA-MB-231-shOLA1 **(B)** compared with their Control. Wnt signaling marker mRNA level was confirmed in MCF-7-PTR **(C)**, and MDA-MB-231-shOLA1 **(D)** compared with their Control. **(E)** Protein level of Smad3, p-Smad3 and Smad 4 in MCF-7-PTR was calculated comparing with MCF-7. **(F)** Protein level of Smad3, p-Smad3 and Smad 4 in MDA-MB-231-shOLA1 was calculated comparing with MDA-MB-231. **(G)** mRNA level Pearson Correlation analysis between OLA1 and TGFB1. Left: Relationship in GTEx Breast mammary tissue and TCGA BRCA normal data. Right: Relationship TCGA BRCA tumor data. **(H)** mRNA level Pearson Correlation analysis between OLA1 and Smad3. Left: Relationship in GTEx Breast mammary tissue and TCGA BRCA normal data. Right: Relationship TCGA BRCA tumor data. **(I)** mRNA level Pearson Correlation analysis between OLA1 and Smad4. Left: Relationship in GTEx Breast mammary tissue and TCGA BRCA normal data. Right: Relationship TCGA BRCA tumor data. (**P < 0.01, ***P < 0.001, Student’s t-test). ns, no significance.

To further investigate the molecular mechanisms of OLA1 regulated EMT resulting in drug resistance to breast cancer, biomarkers in TGF-β/Smad pathway including TGF**β**1, TGF**β**2, SMAD3, and SMAD4 were characterized in the following cell lines: MCF-7 and MCF-7-PTR, and MDA-MB-231 cells with or without OLA1 knockdown. The results showed that TGF-β/SMAD was activated in MCF-7-PTR cells in contrast to MCF-7 (**Figures 6A, E**). Knockdown of OLA1 decreased the expression of TGF-β1, TGF-β2, SMAD4, and SMAD3 significantly (*P* < 0.05) (**Figures 6B, F**), indicating that TGF-β/Smad but not Wnt signaling was inhibited in MDA-MB-231-shOLA1 cells (**Figures 6C, D**). The relationship OLA1 with TGF β 1, SMAD3, and SMAD4 was also validated with Pearson Correlation analysis by GEPIA software. The results showed that the correlation between OLA1 and TGF β 1, SMAD3, and SMAD4 was small, or negatively correlated (**Figures 6G–I**, left) in the breast mammary tissue of the GTEx database and the normal breast tissue of TCGA. However, OLA1 and TGF β 1, SMAD3 SMAD4 has a strong positive correlation in the breast cancer tissue of the TCGA database (**Figures 6G–I**, right). These results indicate that OLA1 deficiency weakens the EMT phenotype through the inhibition of the TGF-β/Smad pathway in either the acquired or intrinsic drug resistant cell lines.

### Mechanism of OLA1 Increases the Resistance of Paclitaxel in Breast Cancer

**Figure 7:**
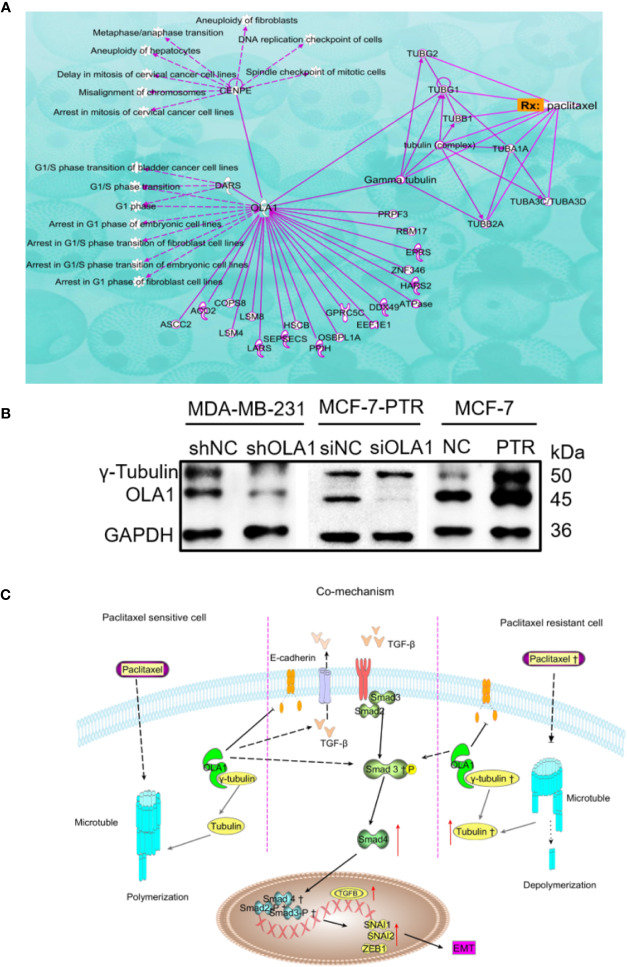
Mechanism of OLA1 increases the resistance of paclitaxel in breast cancer. **(A)** IPA analysis between OLA1 and γ-tubulin in cell cycle regulation. **(B)** OLA1 and γ-tubulin levels in indicated cells. **(C)** Mechanism of OLA1 regulates the resistance in different breast cancer cells.

Ingenuity Pathway Analysis (IPA) was used to clarify the underling the mechanism of paclitaxel resistance regulated by OLA1. As the picture shows (**Figure 7A**), γ-tubulin acts as a bridge linking OLA1 to paclitaxel. We also tested the expression of γ-tubulin in drug-resistant cell lines by knocking down the expression of OLA1. The results showed that the expression of γ-tubulin decreased significantly with the weakening of OLA1 (**Figure 7B**). Our study provides a novel insight that reveals the role of OLA1 in tamoxifen and paclitaxel resistant breast cancer. The mechanism of OLA1 participation in the resistance of paclitaxel in breast cancer was drawn (**Figure 7C**).

## Discussion

OLA1 belongs to the YchF subfamily p-Loop GTPase. The YchF family protein structure mainly includes three domains: N-terminus G domain, the coiled-coil domains on both sides, and the C-terminus TGS domain (ThrRS, GTPase, Spot). OLA1 is highly conserved compared with other Obg family members, but the NKxD consensus sequence in the G4 domain is replaced by NxxE, so it lacks the specificity of binding nucleotides, so it can hydrolyze both ATP and GTP. OLA1 also plays a vital role in cell signal transduction, intracellular transport, cell stress and embryonic development, and protein translation. As an enzyme that also has transport functions and hydrolyzes ATP activity, we can easily associate the influential role played by members of the ABC protein superfamily in tumor multidrug resistance research, including ABCB1 (MDR1), ABCC1, ABCG, etc. Through bioinformatics analysis, we found that overexpression of OLA1 has been found for various types of cancer, including breast cancer, and may be connected with poor survival, (Zhang et al., 2009a; Zhang et al., 2009b; Sun et al., 2010; Bai et al., 2016; Ding et al., 2016; Huang et al., 2020). Therefore, let us justify the hypothesis that OLA1 may be associated with breast cancer resistance.

Tumor metastasis is an important reason for cancer multidrug resistance. EMT plays a consequential role in the process of tumor invasion, and propels cancer cells increased tumor-initiating and metastatic potency with a stubborn resistance to elimination by multiple therapies (Dongre and Weinberg, 2019). Conversely, some chemotherapeutic drugs, while killing cancer cells, also promote the metastasis of cancer cells (Chang et al., 2017; Karagiannis et al., 2017; Keklikoglou et al., 2019). For example, George Karagiannis et al. found that paclitaxel can change the tumor metastasis microenvironment and promote breast cancer metastasis (Karagiannis et al., 2017). Recently, Ioanna Keklikoglou found that the commonly used chemotherapy drugs paclitaxel and doxorubicin can promote the release of exosome by tumors, change the microenvironment in the lung, and promote lung metastasis in breast cancer (Keklikoglou et al., 2019). The loss of the epithelial phenotype and the acquisition of interstitial characteristics are the main features of EMT occurrence.

According to reports, decreased OLA1 expression weakens breast cancer cell motility and invasion (Zhang et al., 2009b). We constructed a paclitaxel-resistant cell line MCF-7-PTR, and found that the drug-resistant cell line promotes the EMT process and OLA1 was highly expressed. The resistance cell MCF-7-PTR with morphology change is associated with the morphology of MCF-7-ADR which is induced by continuous concentration of Adriamycin, and indicated that the resistant cell line may have much heterogeneity. EMT-transcription factors (EMT-TFs) such as TWIST1, SNAIL1, SLUG, and ZEB1, can trigger the EMT process by either directly or indirectly restraining E-cadherin expression (Graham et al., 2008; Taube et al., 2010). Moreover, OLA1 contributes to EMT in lung cancer by modulating the GSK3β/Snail/E-cadherin signaling. OLA1-knockdown cells are more resistant to TGFβ1-induced EMT in A549 cell line. The TGF-β signaling has a proven role in expediting EMT by down-regulating E-cadherin *via* certified EMT-TFs such as TWIST1, SNAIL1, and SLUG (Mallini et al., 2014). Knockdown of OLA1 in lung adenocarcinoma cells can attenuate the TGF-β-induced EMT process and restore E-cadherin expression (Bai et al., 2016). Knockdown of OLA1 caused Egr1, a regulator of oxidative stress, to be down-regulated as well as Smad, and could reverse the process of mouse embryo fibroblast transformation induced by a metal mixture (Martinez-Baeza et al., 2016). Meanwhile, EGR1 is a TGF-β/Smad target that up-regulates the expression of collagen genes and undertakes a crucial role in regulating TGF-β stimulation (Chen et al., 2006). Therefore, this evidence leads us to believe that the chemoresistance of breast cancer cells caused by OLA1 may be achieved through the TGF-β/Smad pathway. In our results, we demonstrated that OLA1 is a positive regulator of the EMT process regardless in the paclitaxel acquired resistance (MCF-7-PTR) cell or endogenous drug resistance cell (MDA-MB-231) in breast cancer. Knockdown of OLA1, TGFB1, TGFB2, SMAD3, and SMAD4 were down regulated accordingly, and p-Smad3 and Smad4 were decreased as well. While in the MCF-7-PTR, in which OLA1 was highly expressed, it had converse results. That is to say, OLA1 can activate the TGF-β/Smad pathway to induce the EMT process in breast drug resistance cells. Bcl-2 has been proven to be an anti-apoptotic protein and is overexpressed in multiple malignant tumors. Bcl-2 inhibits apoptosis by binding to Bax and blocking Bax oligomerization. In our study, in either acquired drug-resistant or intrinsic resistant cell lines, knockdown of OLA1 can cause a decrease in Bcl-2 expression and increase of Bax and cleaved caspase3, indicating improvement of the chemosensitivity of breast cancer.

MCF-7 is an ER-positive breast cancer cell line that is relatively sensitive to tamoxifen in clinical treatment. However, as the process toward the treatment progresses, it is easier to produce tolerance to chemotherapeutics. MDA-MB-231 is a type of triple-negative breast cancer cell line with negative expression of estrogen receptor (ER), progesterone receptor (PR), and human epidermal growth factor receptor 2 (Her2), and usually as endogenous resistant breast cancer cells. This type of breast cancer is prone to greatly metastasize and to endogenous resistance, which undoubtedly brings great difficulties to the treatment of breast cancer. Tamoxifen-resistant breast cancer cells highly overexpress BARD1 and BRCA1, resulting in chemoresistance to DNA-damaging therapies including cisplatin and doxorubicin, but not to paclitaxel (Zhu et al., 2018). This suggests that microtubule-targeting drugs may be given more priority to DNA-damaging agents for treating tamoxifen-resistant breast cancer patients (Zhu et al., 2018). In addition, microtubule-targeting drugs such as paclitaxel, despite their side effects, are still considered the standard therapy against triple negative breast cancer (Anderhub et al., 2019). However, it must not be ignored that some cancer cells will “seek away” slyly and cause patients to become resistant to treatment. Therefore, we want to further study the mechanism of paclitaxel resistance by constructing a paclitaxel-resistant cell line for breast cancer and accumulate therapeutic evidence in the treatment of tamoxifen-resistant breast cancer. Interestingly, in knock down OLA1 expression, the proliferation of MDA-MB-231-shOLA1 was decreased, and cell cycle G1/S marker CCND1 was reduced significantly. From the cell cycle analysis carried by flow cytometry, the MDA-MB-231-shOLA1 in the experimental group was blocked at the G1/S phase. While acquired resistance cell line MCF-7-PTR had no significant changes both in proliferation and cell cycle.

MCF-7-PTR is a drug-resistant cell line induced by paclitaxel. Paclitaxel resistance may be a key factor that causes the difference between the two phenotypes of cells. The breast and ovarian cancer-specific tumor suppressor BRCA1, along with its heterodimer partner BARD1, plays a critical role in DNA repair, drug resistance, centrosome regulation, and transcription (Matsuzawa et al., 2014). OLA1 directly interacted with BARD1, BRCA1, and γ-tubulin in centrosomal regulation (Matsuzawa et al., 2014; Yoshino et al., 2018). Further, tamoxifen-resistant breast cancer cells express observably more BARD1 and BRCA1, lending chemoresistance to DNA-damaging therapy especially in ER-positive breast cancer patients (Zhu et al., 2018). Therefore, we believe that OLA1 may be involved in paclitaxel resistance due to the interaction with γ-tubulin in breast cancer. IPA was used to clarify the underling mechanism of paclitaxel resistance regulated by OLA1. As the picture shows (**Figure 7A**), γ-tubulin acts as a bridge linking OLA1 to paclitaxel, which further shows that our guess is reasonable. OLA1 participates in normal spindle assembly and in the cell cycle regulation process (Xie et al., 2020). The typical centrosome is considered to be the center of microtubule organization and is necessary for spindle assembly (So et al., 2019). Centrosome-associated protein E (CENPE) accumulates in the G2 phase of the cell and is involved in microtubule depolymerization activity near the centromere region. OLA1 can directly interact with CENPE and participate in the G1/S cycle process of various cells, which fully illustrates that OLA1 does participate throughout the process of paclitaxel-mediated resistance to Anti-tubulin. Simultaneously, we tested the expression of γ-tubulin in drug-resistant cell lines by knocking down the expression of OLA1. The results showed that the expression of γ-tubulin decreased significantly with the weakening of OLA1 (**Figure 7B**). OLA1 deficiency attenuates tubulin formation and thus regains the sensitivity of paclitaxel in breast cancer.

## Conclusions

Our study provides a novel insight into revealing the distinct role of OLA1 in tamoxifen and paclitaxel resistant breast cancer. Long-term chemotherapeutic agent exposure facilitates translocation of OLA1 to cell membranes, leading to active TGF-β/Smad signaling pathway and accelerating the EMT process. OLA1 target γ-tubulin to depolymerization microtubules and avoid cell cycle block in paclitaxel-resistant cancer cells instead of tamoxifen resistant breast cancer cells. Blockage of OLA1 may be a potential method to improve the survivability of chemoresistant breast cancer patients. However, doubtless further investigations, including *in vivo* animal model studies and prospective clinical observations, are needed.

## Data Availability Statement

All datasets generated for this study are included in the article/**Supplementary Material**.

## Author Contributions

GGu, JL, and XM participated in the study design, performed experiments, analyzed and interpreted data, and wrote the manuscript. YP, BX, and GGa participated in the study design and data analysis. JH and XT contributed to the acquisition of data. JZ, HZ, and HW revised the manuscript critically for important intellectual content. GGu totally revised and edited the manuscript. All authors read and approved the final manuscript.

## Funding

This work was fully supported by the National Natural Science Foundation of China (No. 81770846 and No. 81803024), Hirshberg Foundation for Pancreatic Cancer Research (GX20171003858 and GX20191005878), and Fundamental Research Funds from the Dalian Universities of Technology under Grant No. DUT17ZD308.

## Conflict of Interest

The authors declare that the research was conducted in the absence of any commercial or financial relationships that could be construed as a potential conflict of interest.

The reviewer JG declared a shared affiliation with one of the authors, HW, to the handling editor at the time of the review.

## Abbreviations

BARD1, BRCA1-associated RING domain protein; BRCA1, breast cancer 1; CCND1, Cyclin D1; DMEM, Dulbecco’s modified Eagle’s medium; ECM, Extracellular matrix; EMT, epithelial-mesenchymal transition; EMT-TFs, EMT-transcription factors; ER, Estrogen receptor; FBS, fetal bovine serum; GAPDH, Glyceraldehyde-3-phosphate dehydrogenase; GTEx, Genotype-Tissue Expression; GSEA, Gene set enrichment analysis; Her2, Human epidermal growth factor receptor 2; MMP, matrix metalloproteinase; MTT, 3-(4,5-dimethylthiazol-2-yl)-2,5-diphenyltetrazolium bromide; mRNA, Messenger RNA; OLA1, Obg-like ATPase 1; PBS, phosphate-buffered saline; PCR, Polymerase chain reaction; PR, Progesterone receptor; RACK1, receptor for activated C kinase 1; RT-qPCR, reverse transcription quantitative PCR; SD, standard deviation; ShRNA, Short hairpin RNA; siRNA, small interfering RNA; TCGA, The Cancer Genome Atlas.
