# Supplementary material for: Obg-Like ATPase 1 Enhances Chemoresistance of Breast Cancer via Activation of TGF-β/Smad Axis Cascades
Source: Front Pharmacol. 2020 May 27;11:666. doi: 10.3389/fphar.2020.00666 (PMC7266972; doi:10.3389/fphar.2020.00666)
Supplement: Supplementary file 1 [file DataSheet_1.pdf]

Supplementary Material

Supplementary Figure

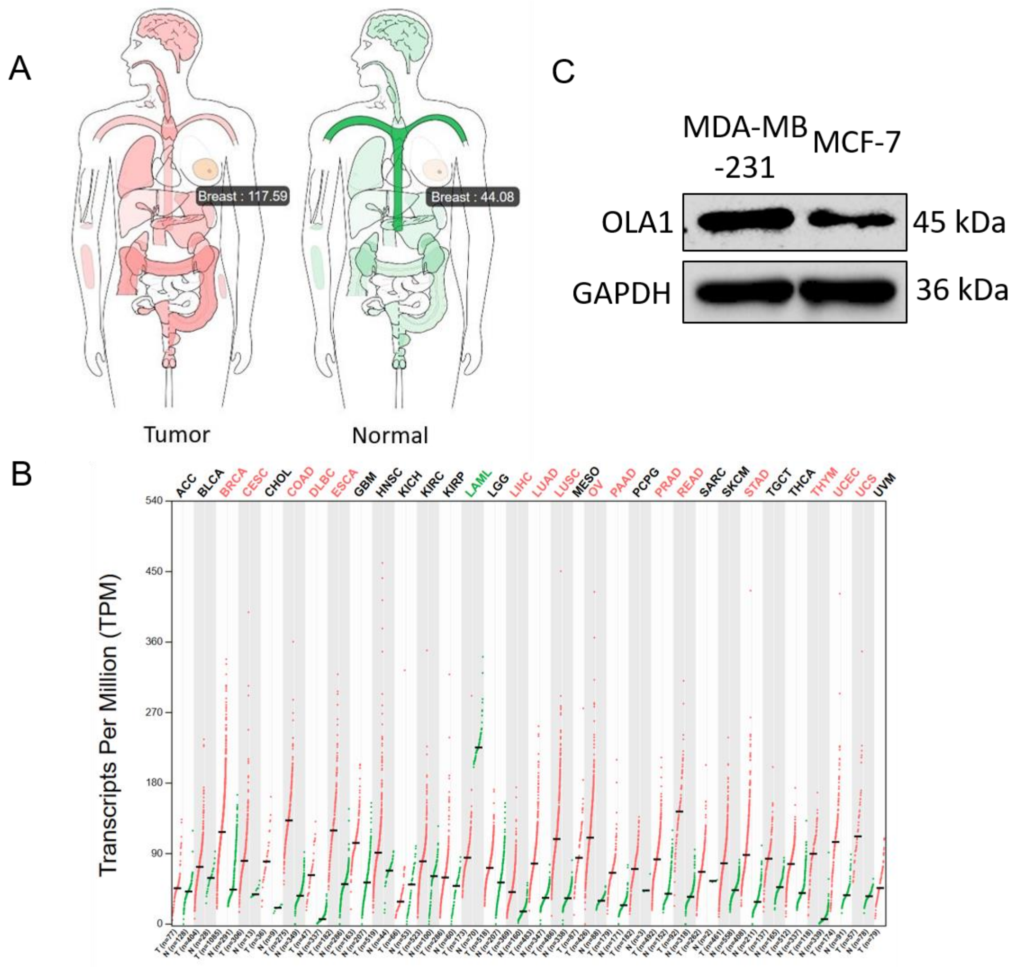

Supplementary Figure 1. OLA1 Enhances Chem-resistance of Breast Cancer via TGFβ/Smad Cascades. **(A)** The median OLA1 expression of tumor (Red) and normal (Green) samples in bodymap. **(B)** OLA1 expression profile across all tumor samples and paired normal tissues. OLA1 high expression (Red), OLA1 down expression (Green), OLA1 expression no significant changes (Black). **(C)** OLA1 expression in MCF-7 and MDA-MB-231 cells.

The tumor abbreviations

ACC Adrenocortical carcinoma

BLCA Bladder Urothelial Carcinoma

BRCA Breast invasive carcinoma

CESC Cervical squamous cell carcinoma and endocervical adenocarcinoma

CHOL	Cholangio carcinoma
COAD	Colon adenocarcinoma
DLBC	Lymphoid Neoplasm Diffuse Large B-cell Lymphoma
ESCA	Esophageal carcinoma
GBM	Glioblastoma multiforme
HNSC	Head and Neck squamous cell carcinoma
KICH	Kidney Chromophobe
KIRC	Kidney renal clear cell carcinoma
KIRP	Kidney renal papillary cell carcinoma
LAML	Acute Myeloid Leukemia
LGG	Brain Lower Grade Glioma
LIHC	Liver hepatocellular carcinoma
LUAD	Lung adenocarcinoma
LUSC	Lung squamous cell carcinoma
MESO	Mesothelioma
OV	Ovarian serous cystadenocarcinoma
PAAD	Pancreatic adenocarcinoma
PCPG	Pheochromocytoma and Paraganglioma
PRAD	Prostate adenocarcinoma
READ	Rectum adenocarcinoma
SARC	Sarcoma
SKCM	Skin Cutaneous Melanoma
STAD	Stomach adenocarcinoma
TGCT	Testicular Germ Cell Tumors
THCA	Thyroid carcinoma
THYM	Thymoma
UCEC	Uterine Corpus Endometrial Carcinoma
UCS	Uterine Carcinosarcoma
UVM	Uveal Melanoma

Supplementary Table

Supplementary Table 1 Primer sequences of indicated genes.

Gene Name	Primer Sequence
**SNAI1-sense**	ACTGCAACAAGGAATACCTCAG
**SNAI1-antisense**	GCACTGGTACTTCTTGACATCTG
**SNAI2-sense**	CACACGGGGGAGAAGCCTTT
**SNAI2-antisense**	ATTGCGTCACTCAGTGTGCT
**VIM -sense**	AGGCAAAGCAGGAGTCCACTGA
**VIM -antisense**	ATCTGGCGTTCCAGGGACTCAT
**ZEB1-sense**	GATGATGAATGCGAGTCAGATGC
**ZEB1-antisense**	ACAGCAGTGTCTTGTTGTTGT
**MMP9-sense**	TTCAGGGAGACGCCCATTTC
**MMP9-antisense**	TGGGTGTAGAGTCTCTCGCT
**TGFB1-sense**	GCAAGTGGACATCAACGGGT
**TGFB1-antisense**	TCCGTGGAGCTGAAGCAATA
**TGFB2-sense**	GGTACCTTGATGCCATCCCGCC
**TGFB2-antisense**	GCACTCTGGCTTTTGGGTTCTGCA
**SMAD3-sense**	TGGACGCAGGTTCTCCAAAC
**SMAD3-antisense**	CCGGCTCGCAGTAGGTAAC
**SMAD4-sense**	GGTTCCTTCAAGCTGCCCTA
**SMAD4-antisense**	ATGTGCAACCTTGCTCTCTCA
**CTNNB1-sense**	GTATGAGTGGGAACAGGGATTT
**CTNNB1-antisense**	CCTGGTCCTCGTCATTTAGC
**MYC-sense**	GTAGTGGAAAACCAGCAGCCT
**MYC-antisense**	TTCTCCTCCTCGTCGCAGTA
**MMP7-sense**	GCATGAGTGAGCTACAGTGGGAAC
**MMP7-antisense**	CCACATCTGGGCTTCTGCATTA
**CCND1-sense**	ATGCCAACCTCCTCAACGAC
**CCND1-antisense**	TCTGTTCCTCGCAGACCTCC
**CDH1-sense**	CGAGAGCTACACGTTCACGG
**CDH1-antisense**	GGGTGTCGAGGGAAAAATAGG
