# Supplementary figures and images for: Obg-Like ATPase 1 Enhances Chemoresistance of Breast Cancer via Activation of TGF-β/Smad Axis Cascades
Source: Front Pharmacol. 2020 May 27;11:666. doi: 10.3389/fphar.2020.00666 (PMC7266972; doi:10.3389/fphar.2020.00666)

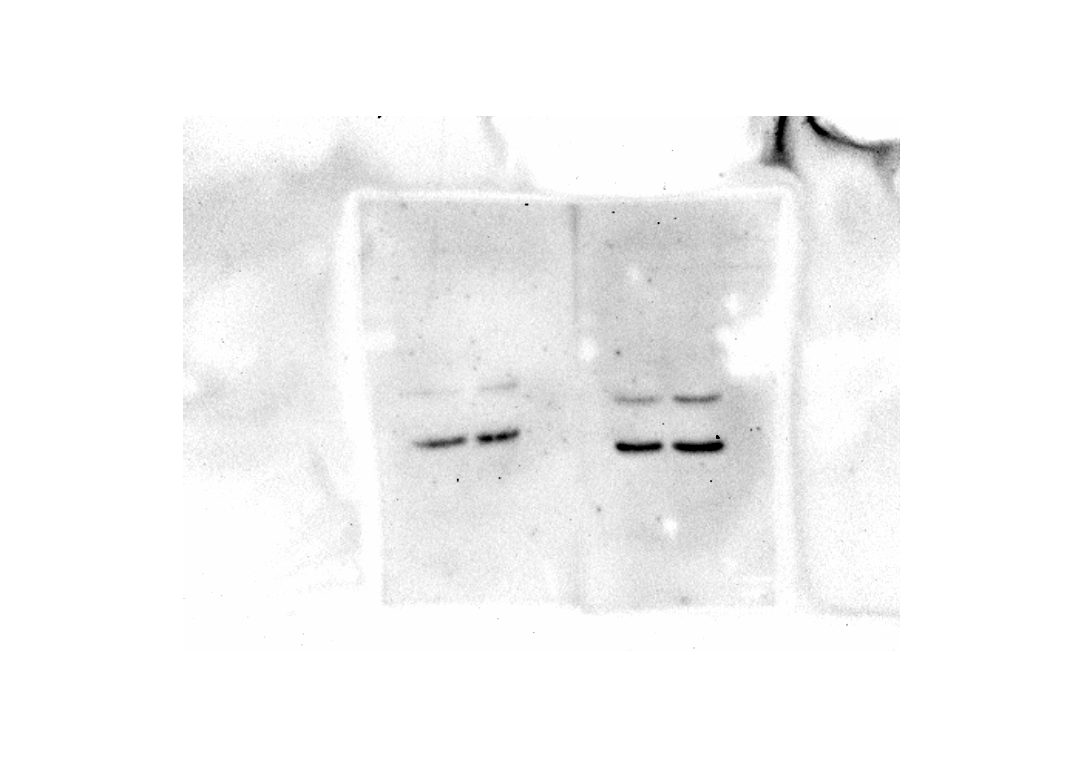

Supplement: Supplementary file 2 [file DataSheet_2.zip › 535966-Blot scans/Fig. 2E.tif]

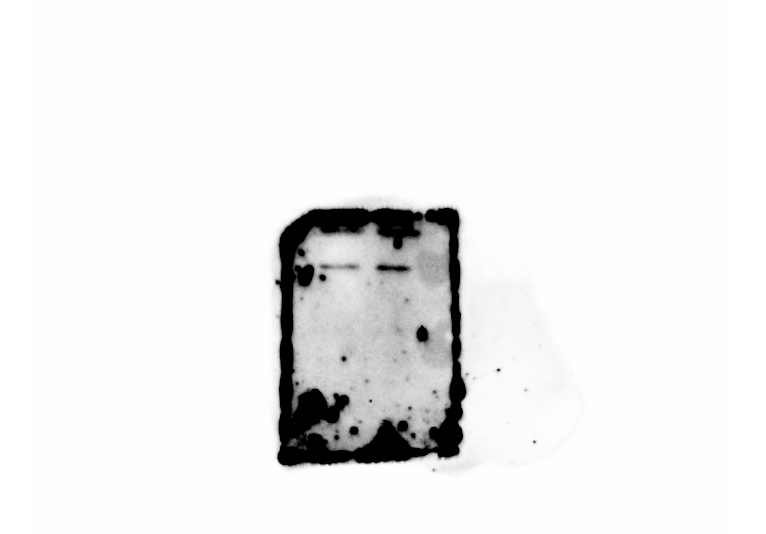

Supplement: Supplementary file 2 [file DataSheet_2.zip › 535966-Blot scans/Fig. 5C-E-cadherin.tif]

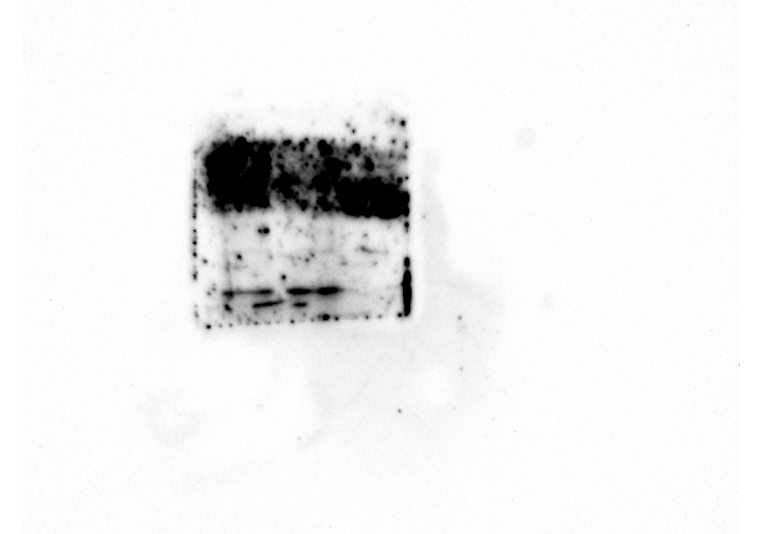

Supplement: Supplementary file 2 [file DataSheet_2.zip › 535966-Blot scans/Fig. 6E-p-smad3.tif]

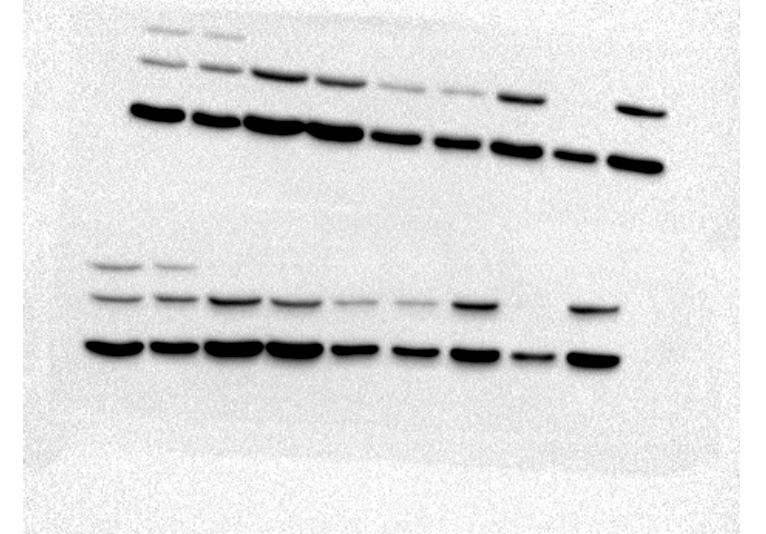

Supplement: Supplementary file 2 [file DataSheet_2.zip › 535966-Blot scans/Fig.3B.tif]

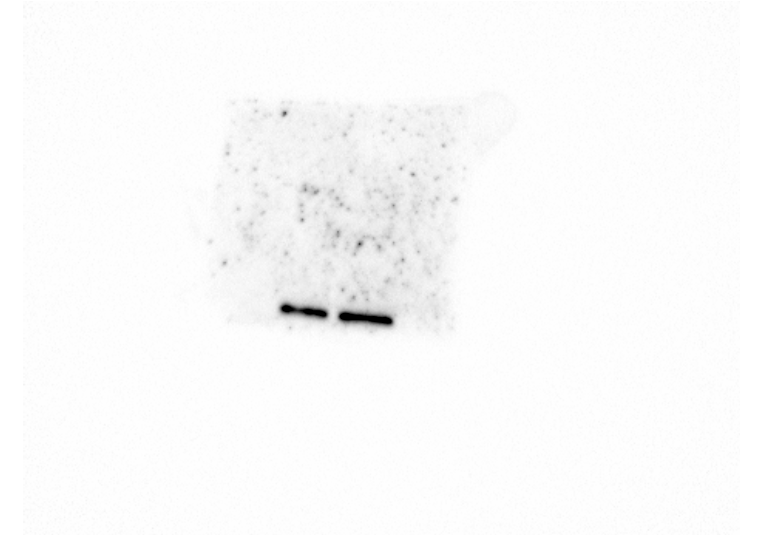

Supplement: Supplementary file 2 [file DataSheet_2.zip › 535966-Blot scans/Fig.3F-BAX.tif]

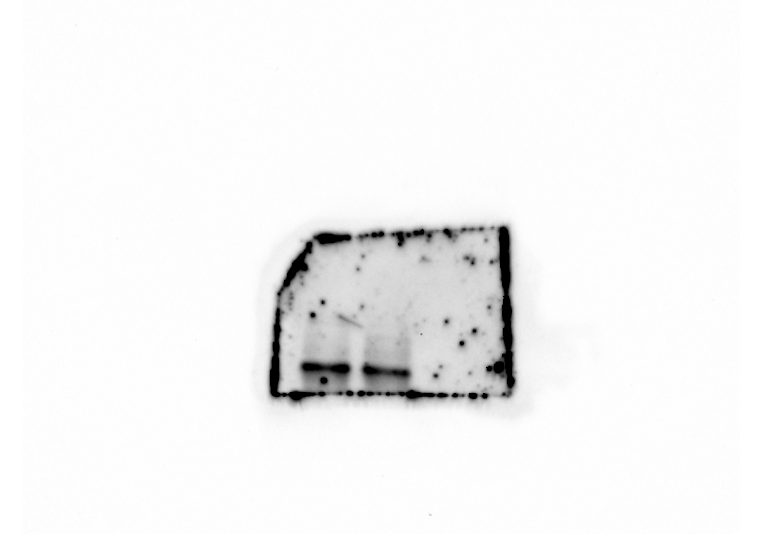

Supplement: Supplementary file 2 [file DataSheet_2.zip › 535966-Blot scans/Fig.3F-BCL2.tif]

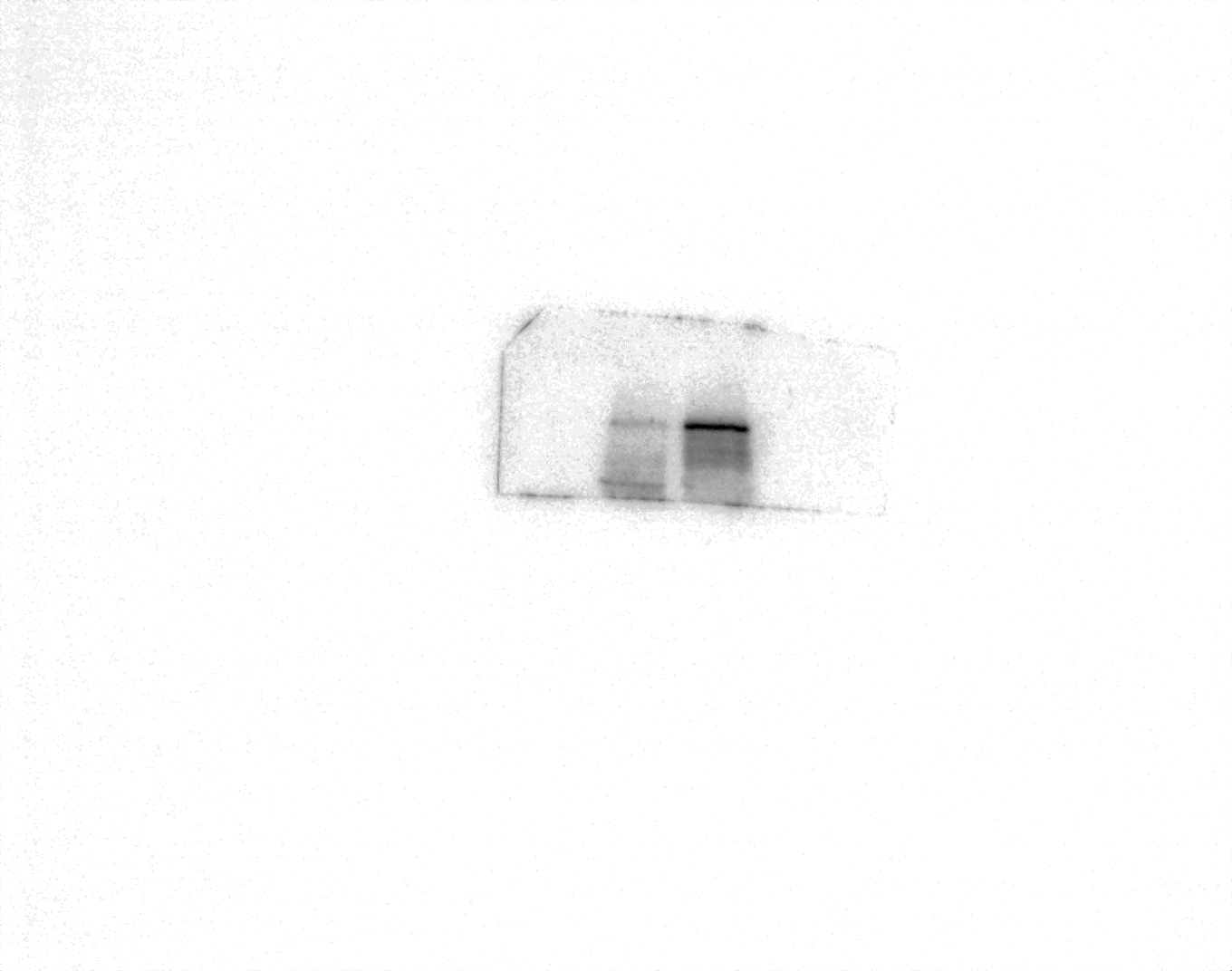

Supplement: Supplementary file 2 [file DataSheet_2.zip › 535966-Blot scans/Fig.3F-Cleaved Caspase 3.tif]

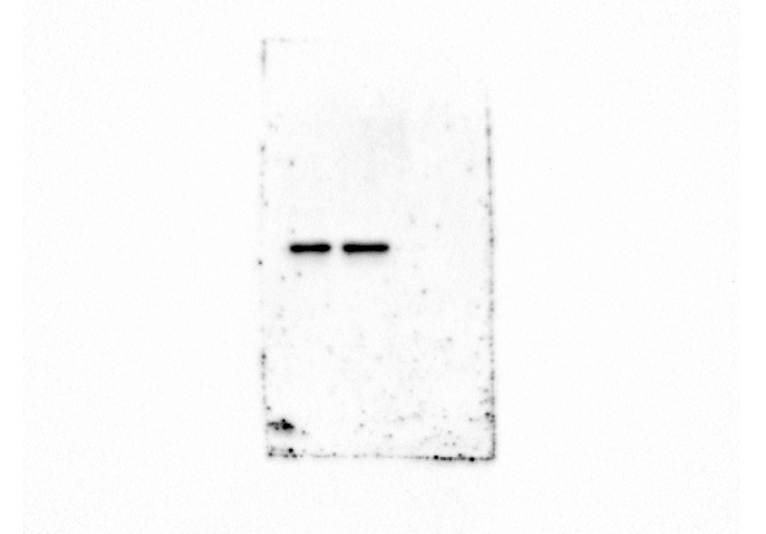

Supplement: Supplementary file 2 [file DataSheet_2.zip › 535966-Blot scans/Fig.3F-GAPDH.tif]

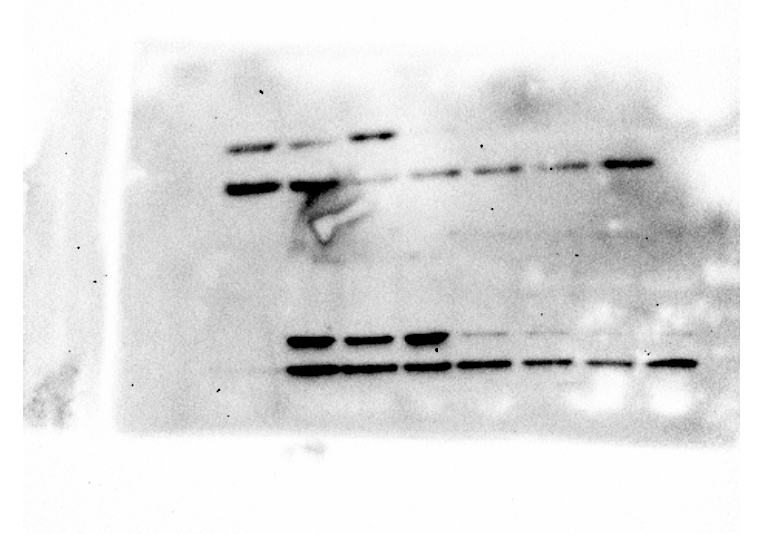

Supplement: Supplementary file 2 [file DataSheet_2.zip › 535966-Blot scans/Fig.4B&4D-OLA1&GAPDH.tif]

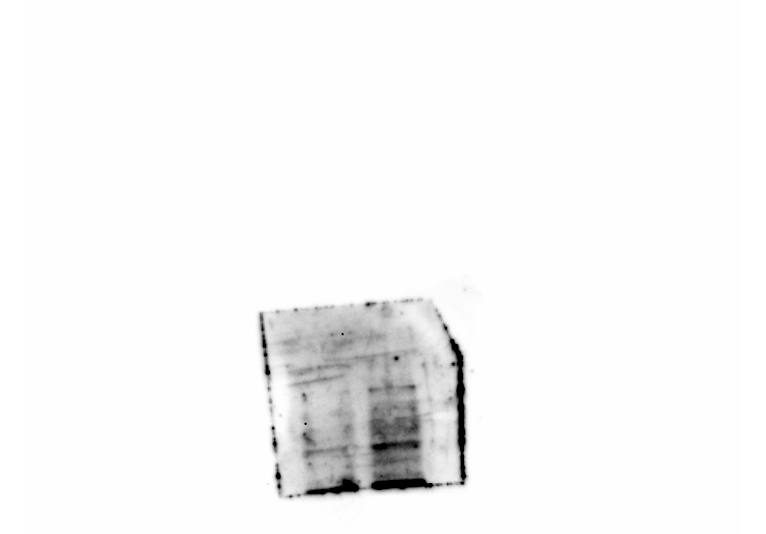

Supplement: Supplementary file 2 [file DataSheet_2.zip › 535966-Blot scans/Fig.4J-Bax.tif]

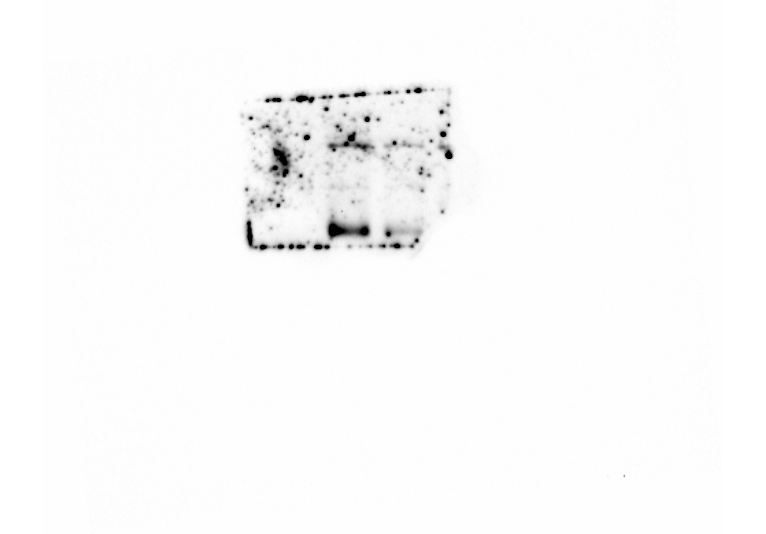

Supplement: Supplementary file 2 [file DataSheet_2.zip › 535966-Blot scans/Fig.4J-Bcl2.tif]

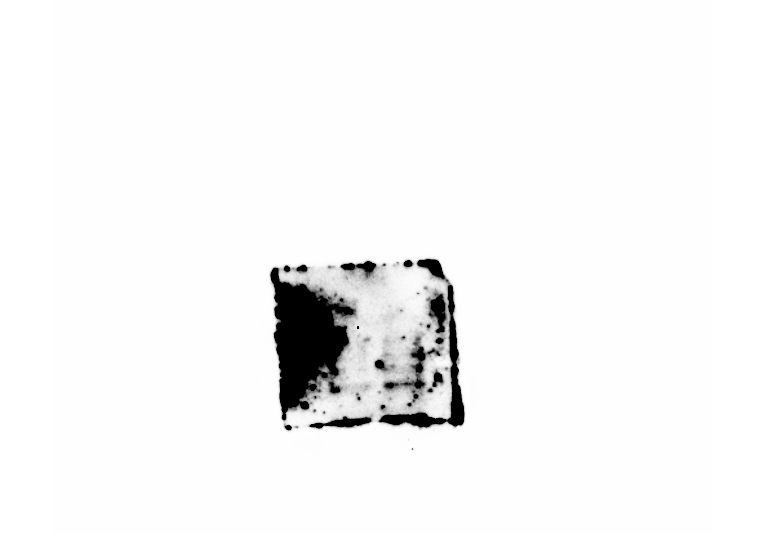

Supplement: Supplementary file 2 [file DataSheet_2.zip › 535966-Blot scans/Fig.4J-Cleaved caspase3.tif]

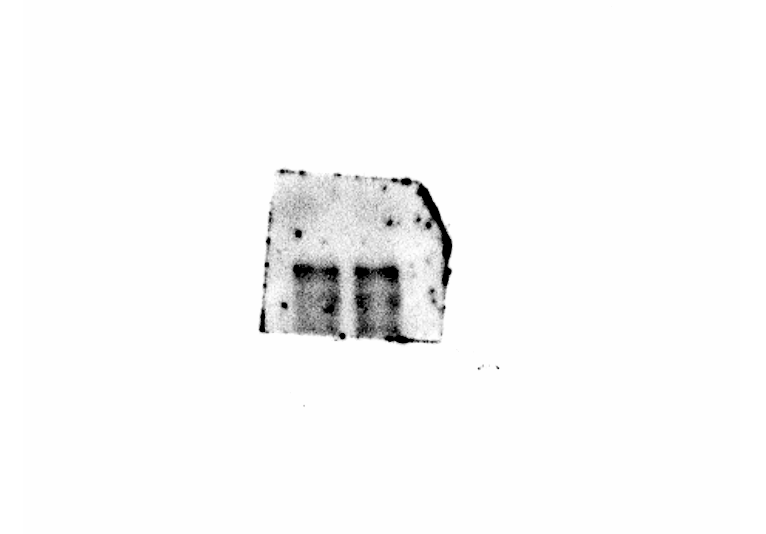

Supplement: Supplementary file 2 [file DataSheet_2.zip › 535966-Blot scans/Fig.4J-GAPDH.tif]

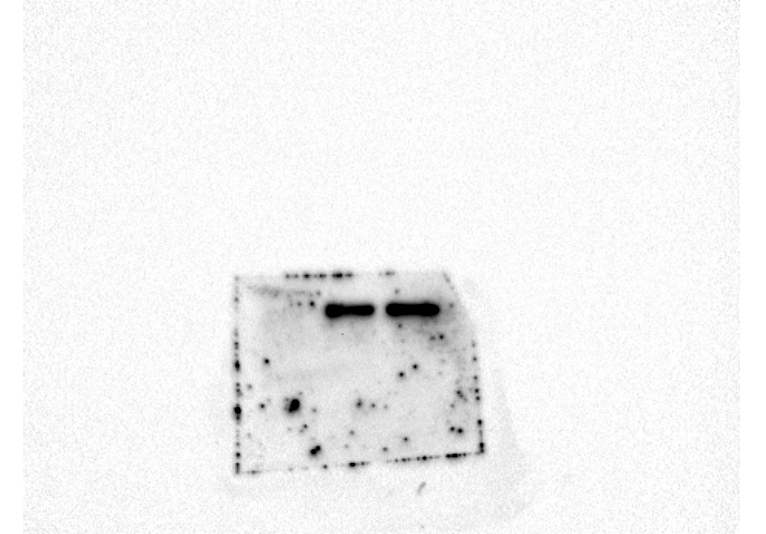

Supplement: Supplementary file 2 [file DataSheet_2.zip › 535966-Blot scans/Fig.5C-GAPDH.tif]

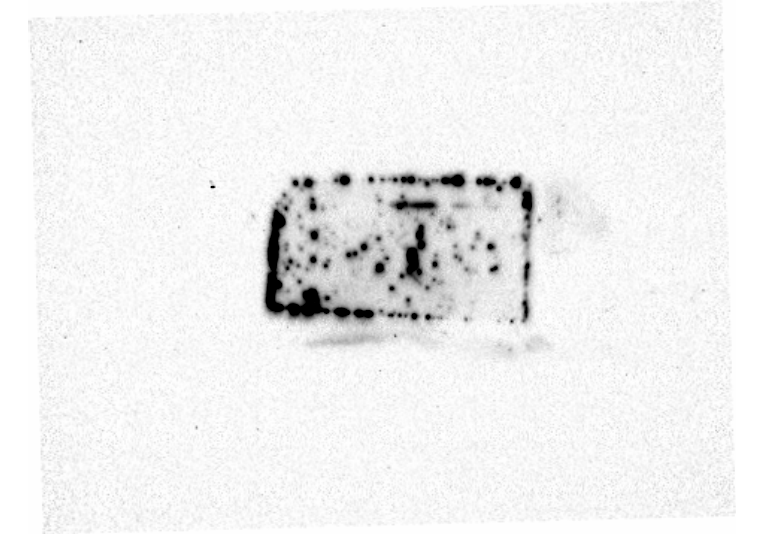

Supplement: Supplementary file 2 [file DataSheet_2.zip › 535966-Blot scans/Fig.5C-SLUG.tif]

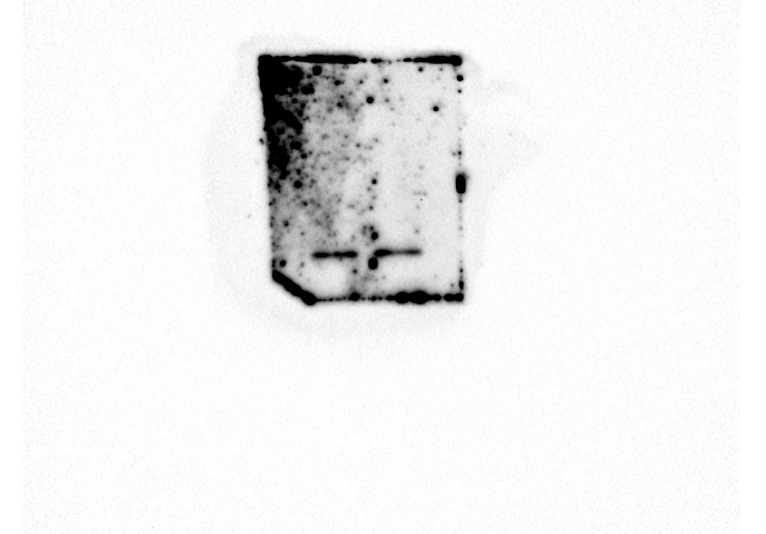

Supplement: Supplementary file 2 [file DataSheet_2.zip › 535966-Blot scans/Fig.5C-Snail.tif]

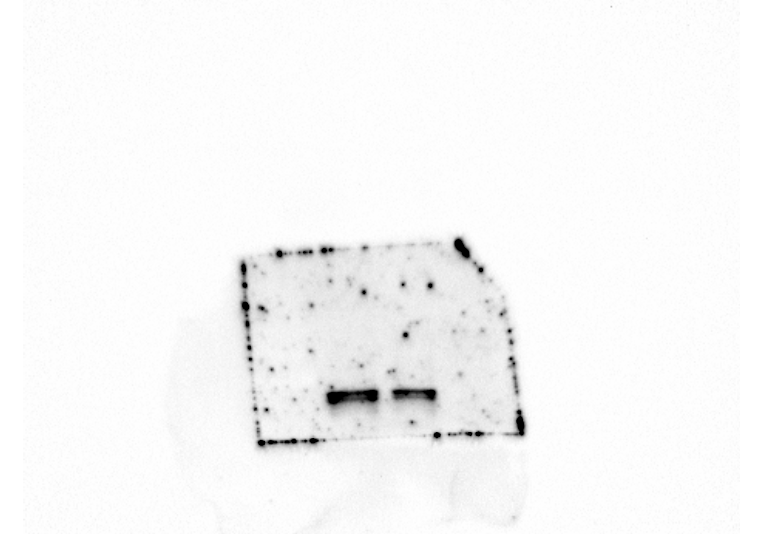

Supplement: Supplementary file 2 [file DataSheet_2.zip › 535966-Blot scans/Fig.5C-Vimentin.tif]

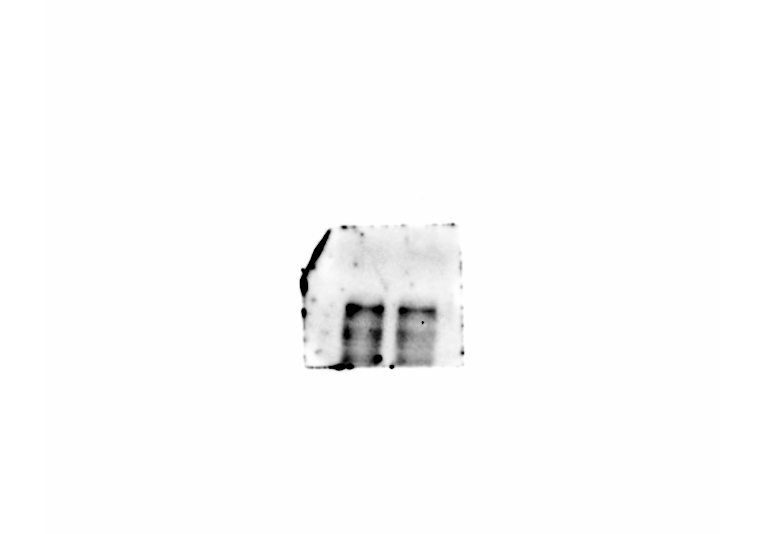

Supplement: Supplementary file 2 [file DataSheet_2.zip › 535966-Blot scans/Fig.5C-ZEB-1.tif]

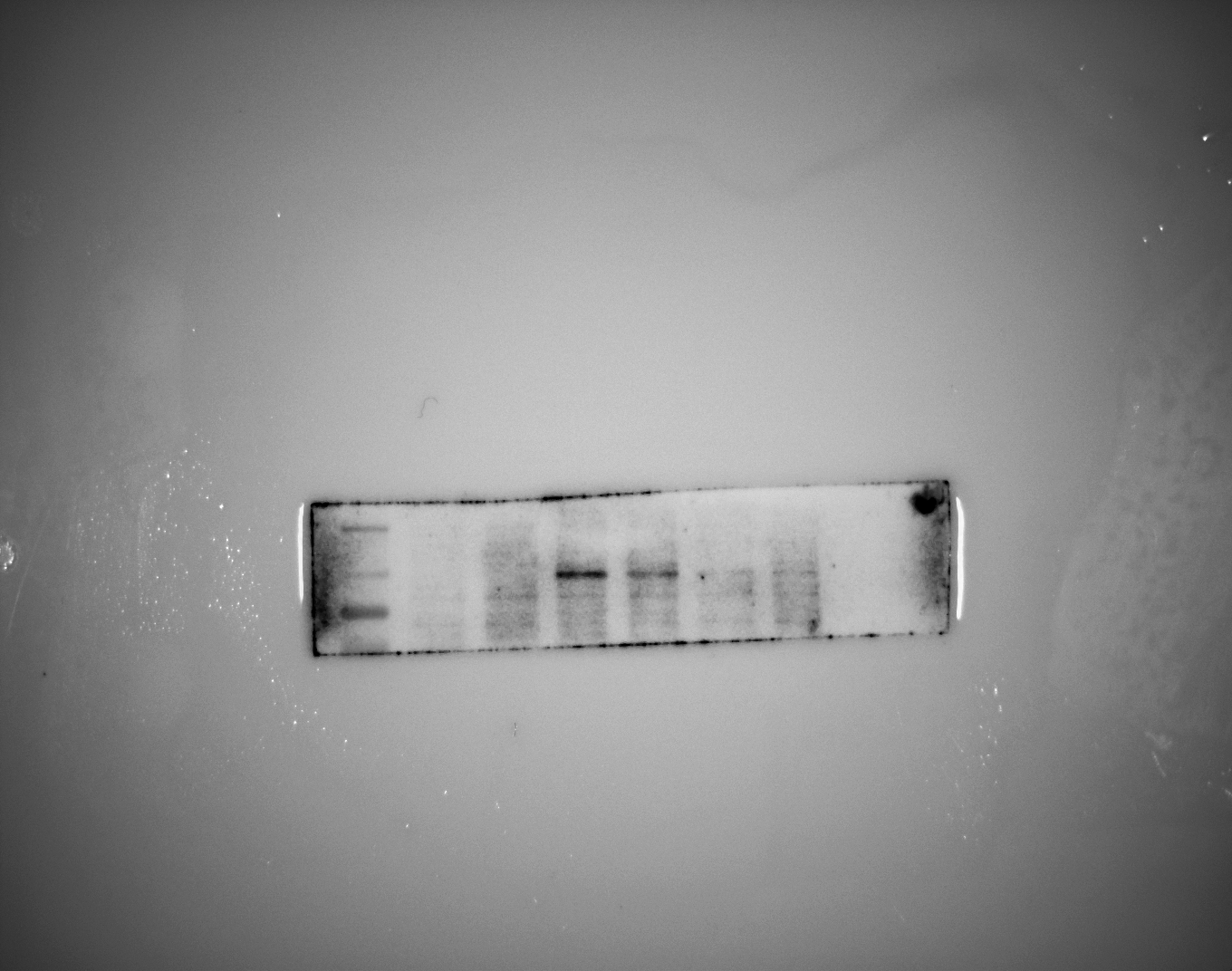

Supplement: Supplementary file 2 [file DataSheet_2.zip › 535966-Blot scans/Fig.5D-E-cadherin.tif]

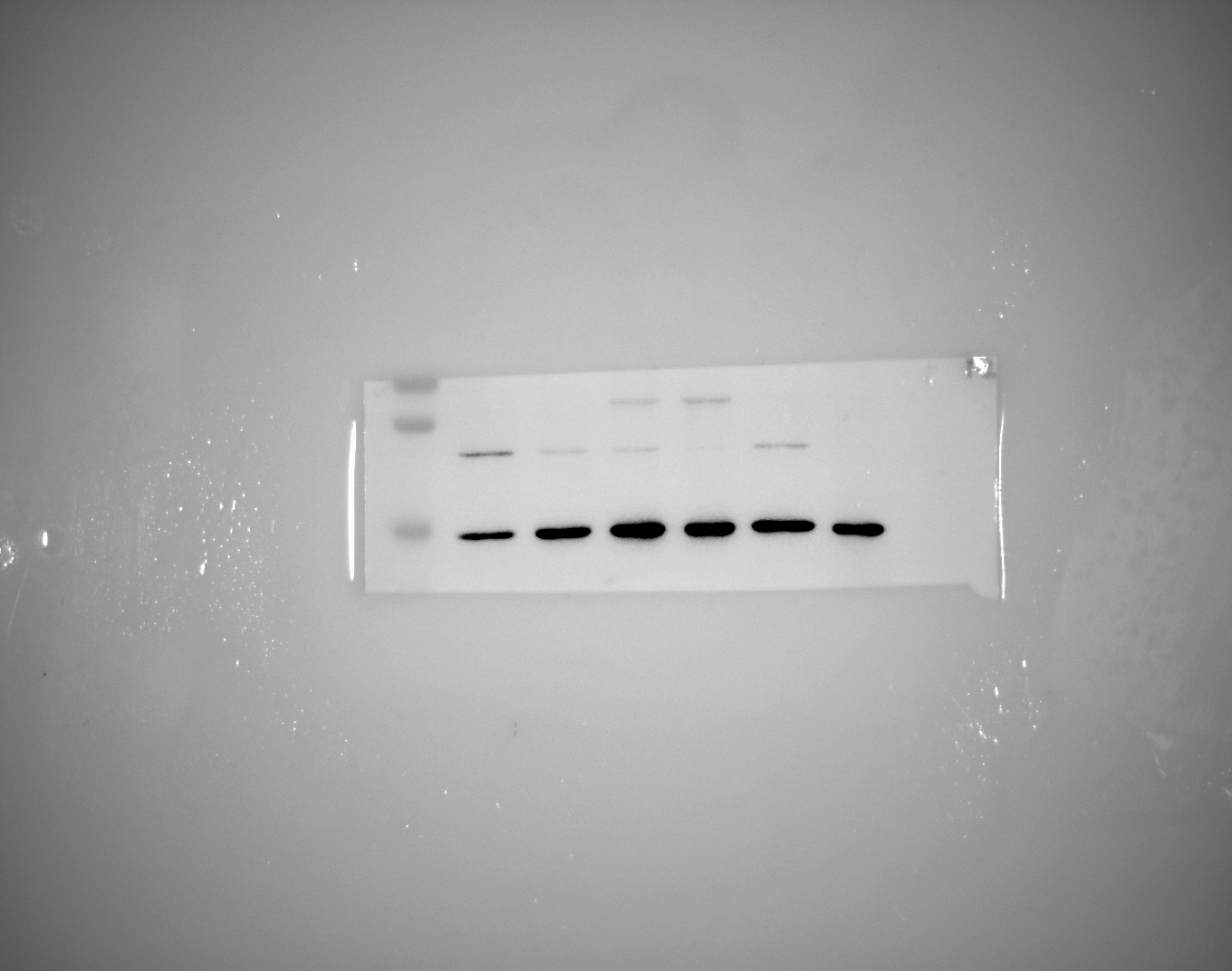

Supplement: Supplementary file 2 [file DataSheet_2.zip › 535966-Blot scans/Fig.5D-GAPDH.tif]

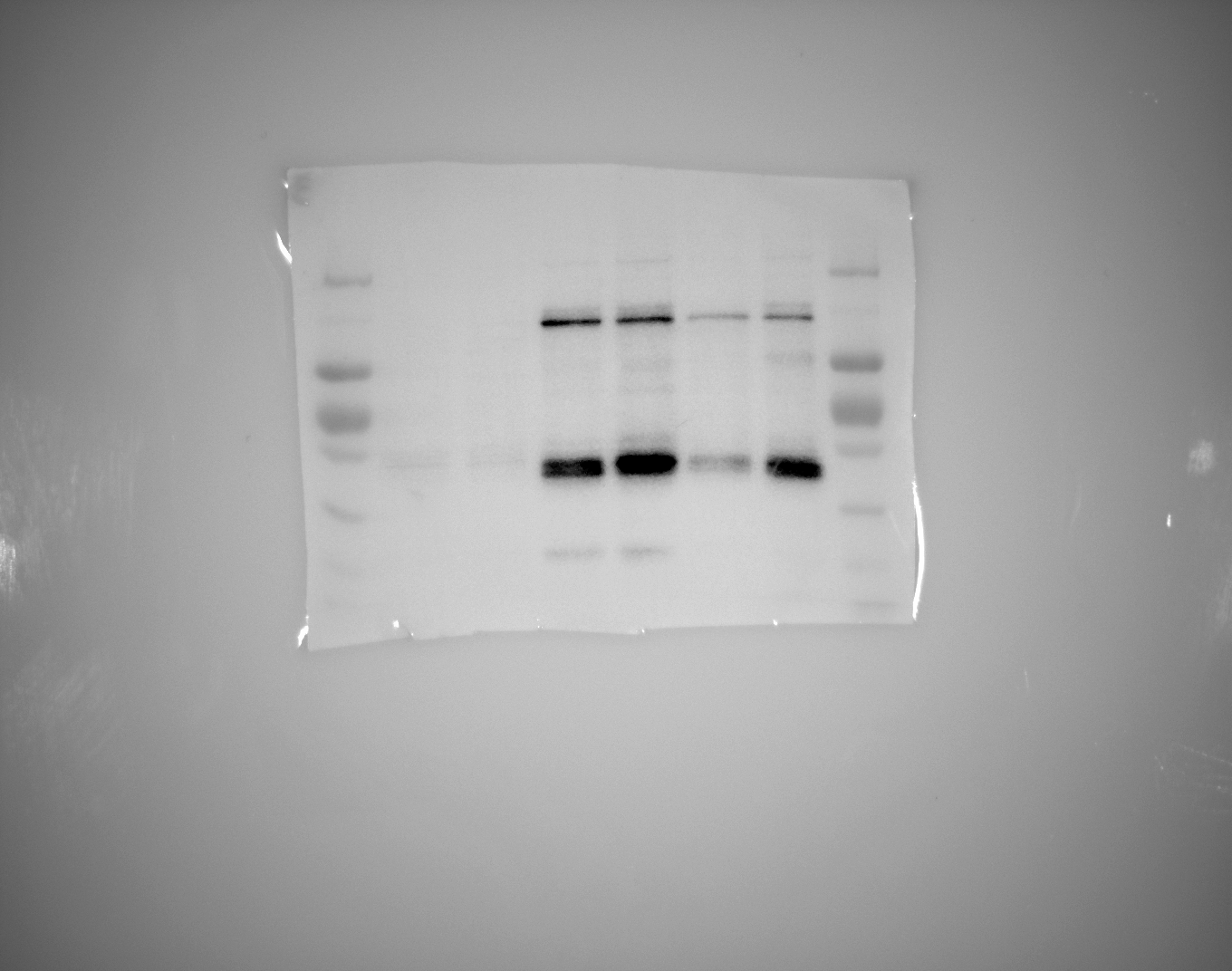

Supplement: Supplementary file 2 [file DataSheet_2.zip › 535966-Blot scans/Fig.5D-snail&slug.tif]

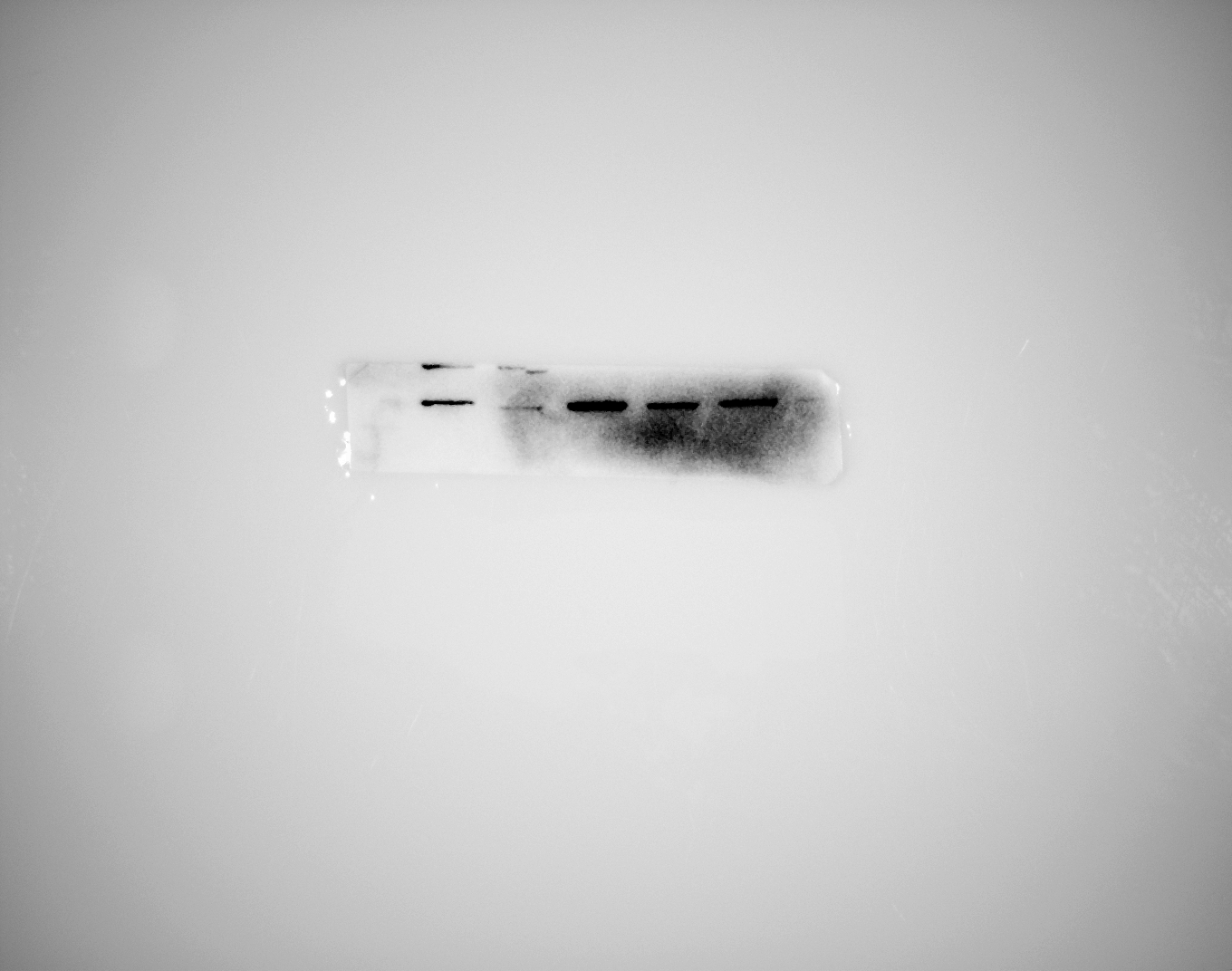

Supplement: Supplementary file 2 [file DataSheet_2.zip › 535966-Blot scans/Fig.5D-Vimentin.tif]

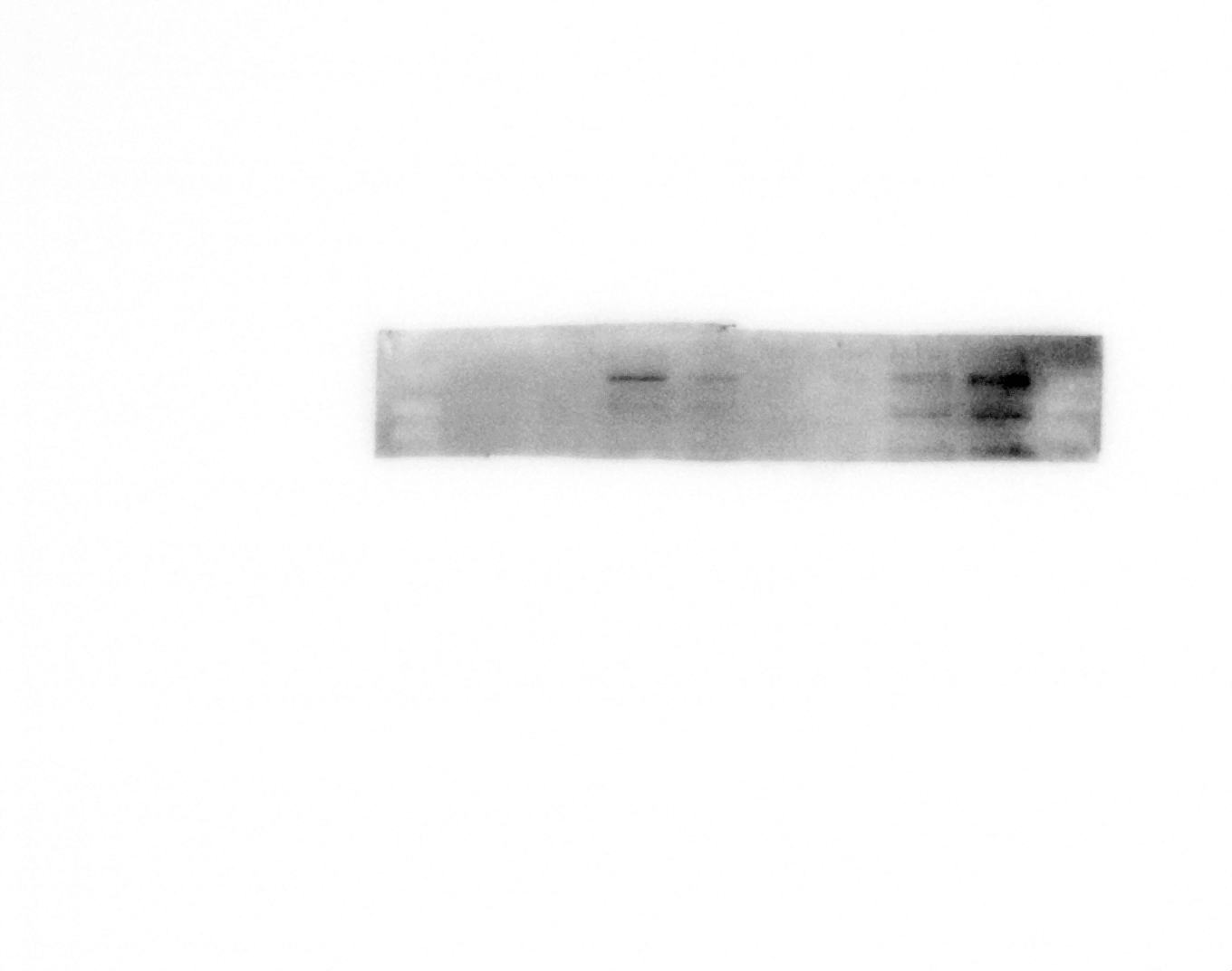

Supplement: Supplementary file 2 [file DataSheet_2.zip › 535966-Blot scans/Fig.5D-ZEB-1.tif]

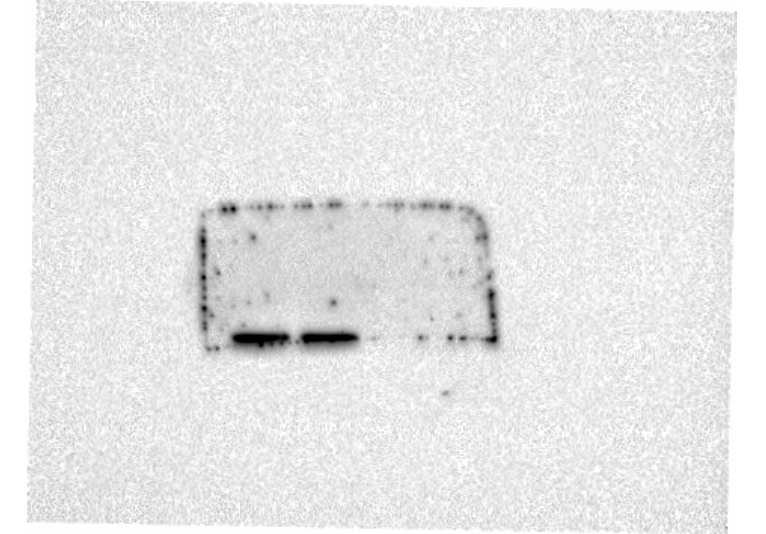

Supplement: Supplementary file 2 [file DataSheet_2.zip › 535966-Blot scans/Fig.6E-GAPDH.tif]

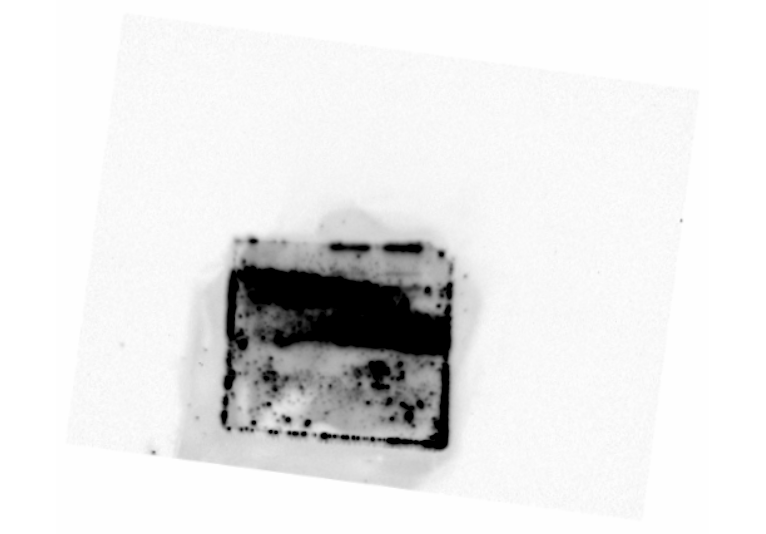

Supplement: Supplementary file 2 [file DataSheet_2.zip › 535966-Blot scans/Fig.6E-Smad3.tif]

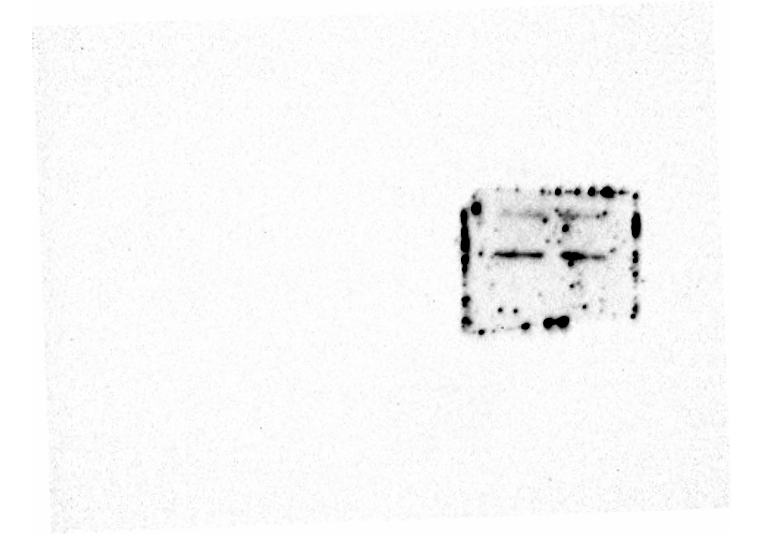

Supplement: Supplementary file 2 [file DataSheet_2.zip › 535966-Blot scans/Fig.6E-SMAD4.tif]

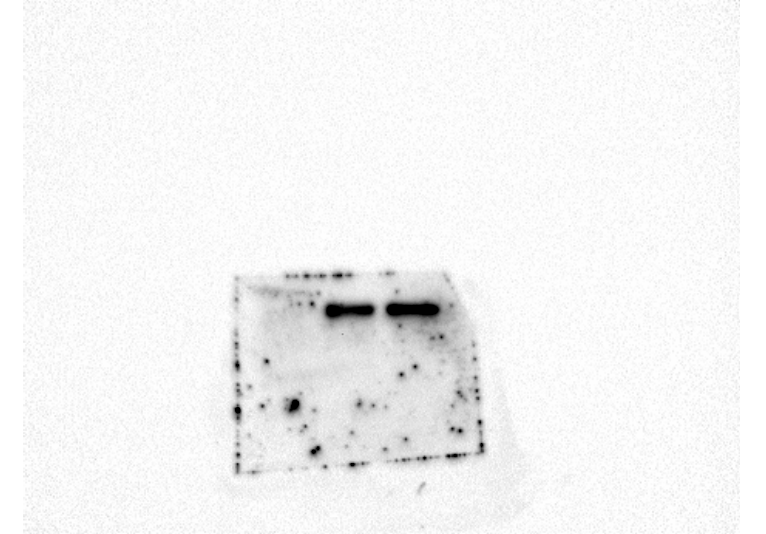

Supplement: Supplementary file 2 [file DataSheet_2.zip › 535966-Blot scans/Fig.6F-GAPDH.tif]

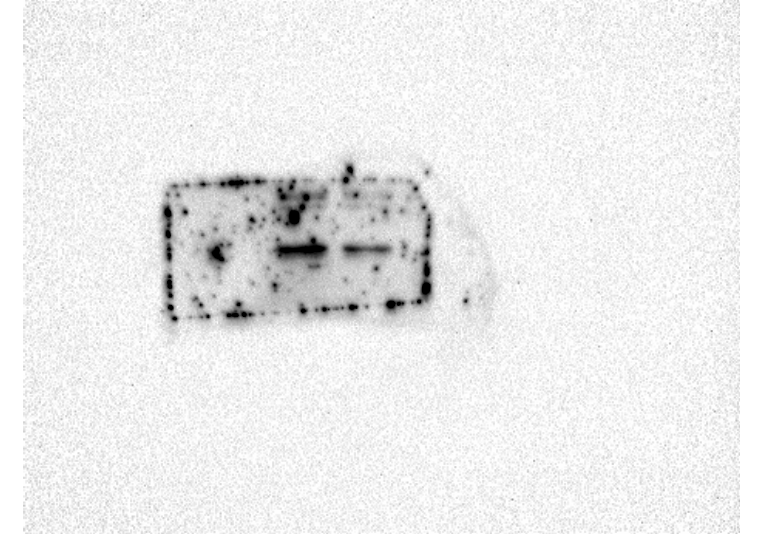

Supplement: Supplementary file 2 [file DataSheet_2.zip › 535966-Blot scans/Fig.6F-p-smad3.tif]

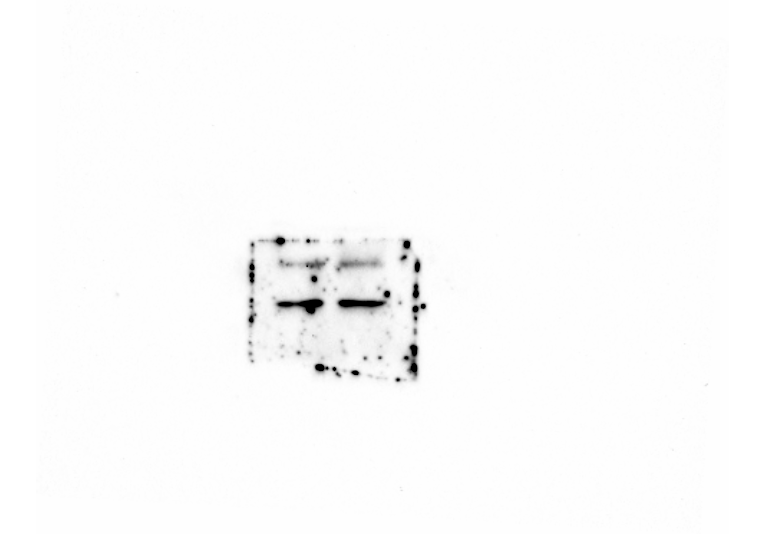

Supplement: Supplementary file 2 [file DataSheet_2.zip › 535966-Blot scans/Fig.6F-SMAD3.tif]

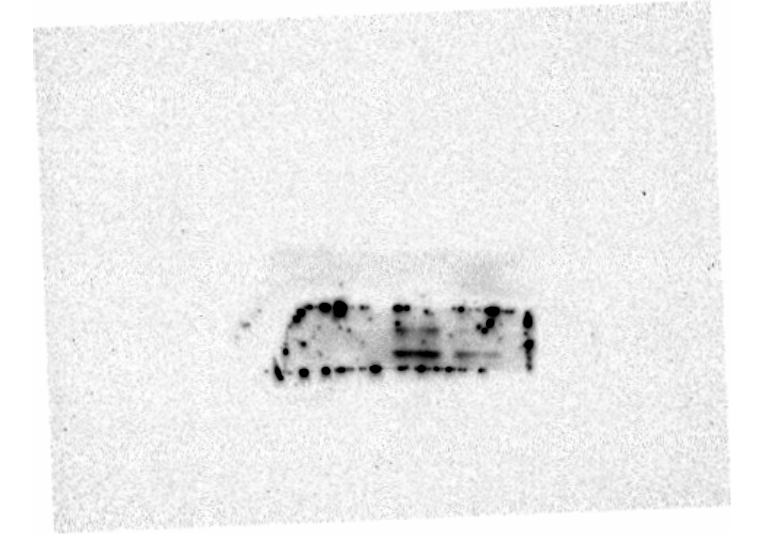

Supplement: Supplementary file 2 [file DataSheet_2.zip › 535966-Blot scans/Fig.6F-SMAD4.tif]

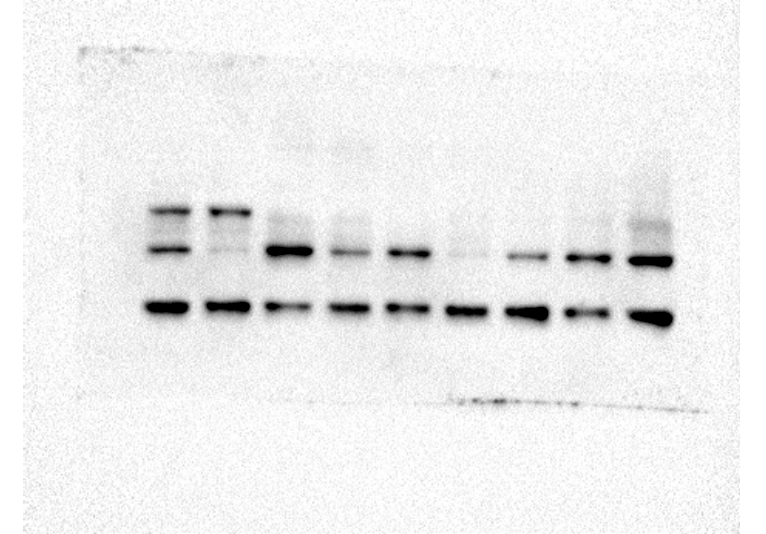

Supplement: Supplementary file 2 [file DataSheet_2.zip › 535966-Blot scans/Fig.7B-MCF-7-PTR.tif]

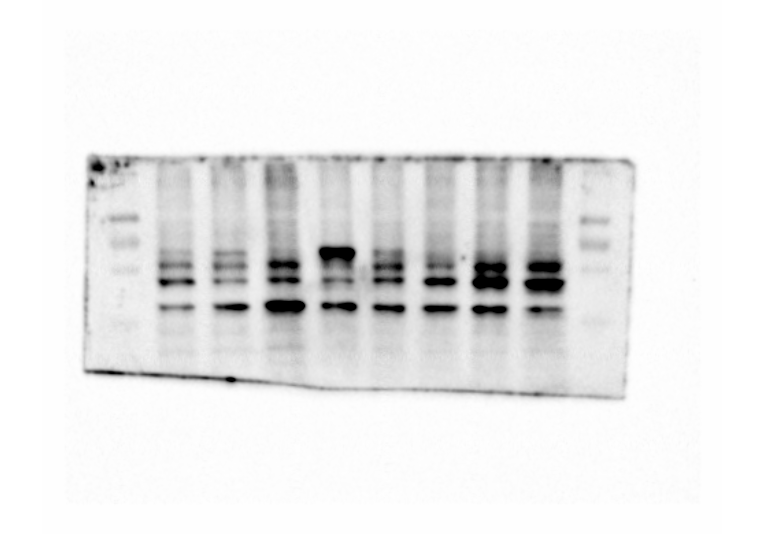

Supplement: Supplementary file 2 [file DataSheet_2.zip › 535966-Blot scans/Fig.7B-MDA-MB-231&MCF-7.tif]
